# Halogen-free layered double hydroxide-cyclotriphosphazene carboxylate flame retardants: effects of cyclotriphosphazene di, tetra and hexacarboxylate intercalation on layered double hydroxides against the combustible epoxy resin coated on wood substrates†

**DOI:** 10.1039/d2ra02586h

**Published:** 2022-08-16

**Authors:** Velusamy Jeevananthan, Swaminathan Shanmugan

**Affiliations:** Department of Chemistry, Faculty of Engineering and Technology, SRM Institute of Science and Technology Kattankulathur 603203 Tamil Nadu India shanmugs2@srmist.edu.in shanmugan0408@gmail.com

## Abstract

The development of halogen-free flame retardants as environmentally friendly and renewable materials for heat and fire-resistant applications in the field of electronics is important to ensure safety measures. In this regard, we have proposed a simple and halogen-free strategy for the synthesis of flame retardant LDH-PN materials to decrease the fire hazards of epoxy resin (EP), *via* a co-precipitation reaction between Mg(NO_3_)_2_ and Al(NO_3_)_3_ and the subsequent incorporation of different cyclotriphosphazene (PN) carboxylate anions. The cyclotriphosphazene-based di, tetra and hexacarboxylate-intercalated layered double hydroxides are designated as LDH-PN-DC, LDH-PN-TC and LDH-PN-HC, respectively. Furthermore, the intercalation of cyclotriphosphazene carboxylate anions into the LDH layers was confirmed by PXRD, FT-IR, TGA, solid-state ^31^P NMR, nitrogen adsorption and desorption analysis (BET), HR-SEM and XPS. Evaluation of the flame retardant (vertical burning test and limiting oxygen index) properties was demonstrated by formulating the LDH-PN materials with epoxy resin (EP) in different ratios coated on wood substrates to achieve the desired behaviour of the EP/LDH-PN composites. Structure–property analysis reveals that EP/LDH-PN-TC-20 wt% and EP/LDH-PN-HC-20 wt% achieved a *V*_0_ rating in the UL-94 V test and achieved higher LOI values (27.7 vol% for EP/LDH-PN-TC-20 wt% and 29 vol% for EP/LDH-PN-HC-20 wt%) compared to the epoxy-coated wood substrate (23.2 vol%), whereas EP/LDH-PN-DC failed in the vertical burning test for various weight percentages of LDH-PN-DC from 5 wt% to 20 wt% in the composites, with a lower LOI value of 22.1 vol%. Excellent flame retardancy was observed for EP/LDH-PN-TC and EP/LDH-PN-HC due to the presence of more binding sites of carboxylate anions in the LDH layers and less or no spiro groups in cyclotriphosphazene compared to that in EP/LDH-PN-DC. In addition, the synergistic flame retardant effect of the combination of LDH and cyclotriphosphazene on the epoxy resin composites remains very effective in creating a non-volatile protective film on the surface of the wood substrate to shelter it from air, absorb the heat and increase the ignition time, which prevents the supply of oxygen during the combustion process. The results of this study show that the proposed strategy for designing flame-retardant properties represents the state-of-the-art, competent coating of inorganic materials for the protection and functionalization of wood substrates.

## Abstract 2

The flame retardant properties of the different types of cyclotriphosphazene carboxylate-intercalated LDH materials are emphasized by increasing the number of binding sites and decreasing the number of spiro groups in the cyclotriphosphazene core.

## Graphical abstract

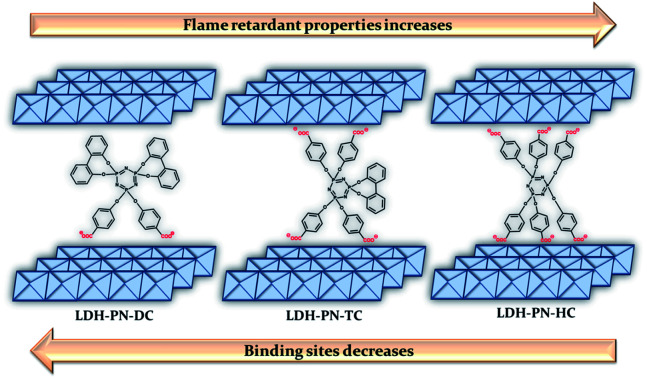

## Introduction

Technical combustion processes are indispensable for the industrialized world to generate power in different forms such as mechanical energy, electrical energy and heat energy. However, fire-related accidents are an impairment of human lives and have social and environmental costs. As a result, combustible materials such as wood, fabrics and synthetic polymers are frequently preserved with flame retardants to suppress fire discharges and tragedies. Flame retardant materials inhibit fire development and propagation, and thereby reduce economic damage and help to protect human lives.^1^ Halogen-containing flame retardant materials display satisfactory fire resistance in a polymer matrix and have been widely used to overcome flammability.^2,3^ Unfortunately, the presence of halogen-containing flame retardant materials may release toxic gases and corrosive fumes during combustion processes, which may cause health and environmental hazards.^4,5^ Therefore, it is important to develop eco-friendly halogen-free intumescent flame retardant materials to enrich the safety measures. Inorganic flame retardants are a fast-growing class of halogen-free flame retardants, which include antimony oxide, magnesium hydroxide and aluminium hydroxide, and the main drawback of these flame retardants is that a very high loading is required to attain satisfactory flame retardant ratings for the final products.^6^

In recent years, layered double hydroxides (LDHs) have received much attention in the synthesis of the halogen-free flame retardants due to their non-toxicity, low cost, high thermal stability, adjustable chemical composition, unique layered structure, and exchangeable interlayer anions.^7,8^ LDHs are layered materials made of positively charged metal hydroxide sheets, negatively charged anions and water molecules at the gallery to balance the charge.^9^ The general chemical formula of LDHs can be illustrated as [M^2+^_1−*x*_ M^3+^_*x*_(OH)_2_]^*x*+^[A^*n*−^_*x*/*n*_]·*m*H_2_O, where M^2+^ and M^3+^ are divalent and trivalent metal cations, respectively, and A^*n*−^ are the interlayer anions. Strong interlayer electrostatic interactions are caused between the LDH sheets due to the high charge density of the LDH layers, high content of anionic species and water molecules representing hydrophilic properties.^10^ Because of these properties, it is desirable that pristine LDHs be altered by the intercalation of different types of organic modifiers to modify their surface and interior properties by weakening the electrostatic forces between the LDH sheets, increasing the interlayer distance of LDHs for the penetration of larger polymers and making the LDHs organophilic.^11^ A wide range of organic modifiers such as carboxylates, phosphonates and sulfonates have been utilized to alter the surface properties of LDHs.^12,13^ Most of the modified LDHs, such as LDH-long-chain linear alkyl carboxylates and sulfonates, are used as flame retardant additives for blending with low-density polyethylene, polypropylene, poly(methyl methacrylate), polystyrene and poly(l-lactide) to yield LDH-polymer nanocomposites.^14,15^ During the combustion process, the flame retardant property of LDH-polymer composites can contribute by absorbing heat, increasing the ignition time, releasing aqueous vapour, reducing combustible gases during pyrolysis and producing an oxide layer on the surface of the material, which can prevent further degradation.^16,17^

Besides organic modifiers in LDHs, inorganic anions such as phosphates and borates are intercalated in LDH layers and used as flame retardant additives as the synergistic effect between the phosphates or borates equally distributed in the interlayer region and the host LDH layers reduces the rate of heat release and the fire growth rate index compared to the LDH carbonate or sulphate precursors.^18,19^ As the mainly used compound, cyclotriphosphazene is a versatile inorganic ring compound in which phosphorous and nitrogen atoms are arranged in alternating positions and two chlorine atoms are attached to each phosphorous atom, which is reactive to different nucleophiles, offering synthetic flexibility to introduce desirable functionalities that can subsequently be transformed into desired synthetic precursors.^20,21^ Cyclotriphosphazene and its derivatives have been commonly used as halogen-free flame retardants owing to their high reactivity, non-toxic nature, high thermal stability, excellent flame retardant efficiency and self-extinguishability that are brought about by their unique molecular design of a PN ring structure.^22–24^ Several substituted cyclotriphosphazenes have been reported so far to develop potential flame retardants with enhanced thermal stability using two different approaches: (i) the first approach is the blending of different substituents on the aryl group of hexaphenoxycyclotriphosphazene with thermosetting polymers^25–29^ and (ii) the second approach is the synthesis of different crosslinked cyclomatrix phosphazene polymers and cyclolinear and spirocyclic phosphazene epoxy resins using cyclotriphosphazene with desired functional groups.^30–36^ Due to the endothermic decomposition of cyclotriphosphazene polymers, the non-volatile protective films on the surface of the polymeric materials are generated to insulate them from air; concurrently, non-flammable gases are also released, which stop the oxygen supply.^37–39^ Recently, the effects of the addition of nickel–iron, nickel–aluminium, and nickel–chromium LDH-sodium dodecyl sulfate (LDH-SDS) and hexaphenoxycyclotriphosphazene (HPCP) on the poly(lactic acid) (PLA) matrix to produce PLA/HPCP/LDH-SDS composites by the melt mixing method have been suggested, and the different types of LDH-SDS materials show the important function of enhancing the thermal stability and flame retardancy of PLA composites by reducing the mass and heat transfer between the gas and condensed phases. Furthermore, the modified nickel–cobalt metal layered double hydroxide with polyphosphazene produces the Ni–Co-LDH@PZS architecture for the application of high fire safety, especially the suppression of smoke and toxic gases during epoxy resin burning.^40^ To the best of our knowledge, there is no report on the intercalation of cyclotriphosphazene carboxylate anions into LDH layers and its application in flame retardancy. Therefore, the combination of both LDH and cyclotriphosphazene through non-covalent bonding will produce LDH-PN materials possessing superior flame retardancy by increasing the interlayer distance of the LDH layers for the dispersion of polymers such as epoxy resin. Based on the above discussion, three cyclotriphosphazene carboxylate anion-intercalated Mg–Al-LDH materials, represented as LDH-PN materials, were synthesized and characterized. Then, the LDH-PN materials were incorporated into epoxy resin with different loadings using ultrasonication and thermal curing processes, which were then coated on a wood substrate. Subsequently, the flame retardancy of the EP/LDH-PN composite-coated wood substrates was estimated using the vertical burning test (UL 94 V) and limiting oxygen index (LOI) test. Excellent flame retardancy was observed for EP/LDH-PN-TC-20 wt% and EP/LDH-PN-HC-20 wt% in the UL 94 V test, and they displayed high LOI values (27.7 vol% for EP/LDH-PN-TC-20 wt% and 29 vol% for EP/LDH-PN-HC-20 wt%), whereas both epoxy resin and EP/LDH-PN-DC-20 wt% failed in the UL 94 V test and showed low oxygen concentration in the LOI test (23.2 vol% for epoxy resin and 22.1 vol% for EP/LDH-PN-DC-20 wt%).

## Experimental section

### Materials and methods

All chemicals were of analytical grade and used without further purification. Hexachlorocyclotriphosphazene (Sigma Aldrich), 2,2′-biphenol (Sigma Aldrich) and methyl 4-hydroxybenzoate (AVRA) were recrystallized from *n*-hexane, dichloromethane and acetone, respectively. Acetone, used as a solvent in the reactions, was pre-distilled with KMnO_4_ and further distilled with anhydrous K_2_CO_3_. Commercially available chemicals such as Al(NO_3_)_3_·9H_2_O (Merck), Mg(NO_3_)_2_·6H_2_O (SRL), 2,2-bis(4-glycidyloxyphenyl) propane (TCI) as epoxy resin, and triethylenetetramine (TETA) (SRL) were used as received. Deionized water was employed in the hydrolysis reactions and LDH synthesis. dispiro-N_3_P_3_(O_2_C_12_H_8_)_2_Cl_2_ (1), spiro-N_3_P_3_(O_2_C_12_H_8_)Cl_4_ (3)^41,42^ and N_3_P_3_(OC_6_H_4_COOH)_6_ (L_3_)^43^ were synthesized according to the reported procedures. The detailed synthetic procedures of [dispiro-N_3_P_3_(O_2_C_12_H_8_)_2_(OC_6_H_4_COOH)_2_] (L_1_) and [spiro-N_3_P_3_(O_2_C_12_H_8_)(OC_6_H_4_COOH)_4_] (L_2_) are given in the ESI.† The Fourier transform infrared (FT-IR) spectra were examined on a Shimadzu IR Tracer-100 in the range of 400 to 4000 cm^−1^. Nuclear magnetic resonance (^1^H, ^13^C and ^31^P) spectroscopy was carried out using a Bruker Advance DPX-250 spectrometer operating at 500 MHz. Electrospray ionization mass spectrometry (ESI-MS) was performed on a Shimadzu LC-MS 2020 spectrometer equipped with an LC10ADVP binary pump. The changes in crystallinity were observed using powder X-ray diffractometry (PXRD, PANalytical India, Spectris Technologies) with Cu Kα radiation (*λ* = 1.54 Å) in the range of 5° to 100°. The thermal stability and weight loss were studied by thermogravimetric analysis (TGA) using a Netzsch-STA 2500 Regulus instrument at a heating rate of 10 °C per min under a nitrogen atmosphere. The morphology of the samples was observed using high-resolution scanning electron microscopy (HR-SEM, Thermosceintific Apreo S). The surface area of the samples was measured by the Brunauer–Emmett–Teller (BET) method on a Quantachrome Autosorb iQ sorption analyzer. The chemical composition was studied by X-ray photoelectron spectroscopy (XPS) using a ULVAC-PHI, PHI5000 Version Probe III, Physical Electronics instrument. The flame retardant properties were examined by the vertical burning test (UL-94 V), and limiting oxygen index (LOI) tests were carried out on a Stanton Red Croft FTT.

### Synthesis of cyclotriphosphazene carboxylate anion-intercalated LDH (LDH-PN)

#### Synthesis of cyclotriphosphazene dicarboxylate anion-intercalated LDH (LDH-PN-DC)

The matched molar ratio of Mg^2+^/Al^3+^/cyclotriphosphazene dicarboxylic acid was 4 : 2 : 1. A solution of Mg(NO_3_)_3_·6H_2_O (7.72 mmol) and Al(NO_3_)_3_·9H_2_O (3.86 mmol) in 20 ml deionized water was slowly added to a 30 ml aqueous solution of NaOH (0.1 N) and cyclotriphosphazene dicarboxylic acid (L_1_) (1.93 mmol) with vigorous stirring under N_2_ atmosphere, and the value of the pH was adjusted to above 10 by adding a 1 M NaOH solution. The resulting slurry was aged at 70 °C for 36 h, centrifuged, washed with deionized water until pH = 7.0, and dried at 70 °C for 2 days. The final dried solid was crushed with a mortar and pestle to produce a fine powder. Yield: 2.2 g. IR (ATR, cm^−1^): 3420(b), 1599(s), 1535(s), 1499(m), 1474(m), 1438(m), 1384(s), 1272(s), 1231(s), 1216(m), 1160(s), 1093(s), 937(s), 885(s), 780(s), 749(s), 717(m), 665(s), 604(s), 563(m), 540(m). Solid-state ^31^P NMR (162 MHz) *δ* (ppm): 29.0 ([C_12_H_8_O_2_]_2_, 2P), 10.4 ([C_7_H_4_O_3_]_2_, 1P). TGA: temperature range (weight loss): 70–215 °C (9.2%), 230–720 °C (40.9%) and 800–1000 °C (4.7%).

#### Synthesis of cyclotriphosphazene tetracarboxylate anion-intercalated LDH (LDH-PN-TC)

LDH-PN-TC was prepared by a similar synthetic procedure as LDH-PN-DC using cyclotriphosphazene tetracarboxylic acid (L_2_) instead of cyclotriphosphazene dicarboxylic acid (L_1_). The matched molar ratio of Mg^2+^/Al^3+^/cyclotriphosphazene tetracarboxylic acid (L_2_) was 8 : 4 : 1 (13.83 mmol : 6.92 mmol : 1.73 mmol). Yield: 2.1 g. IR (ATR, cm^−1^): 3401(b), 1599(s), 1555(s), 1544(m), 1500(m), 1384(s), 1271(m), 1212(m), 1160(s), 1159(s), 1095(s), 1013(m), 970(s), 883(m), 780(m), 754(b), 665 (s), 606(s), 553(s). Solid-state ^31^P NMR (162 MHz) *δ* (ppm): 28.0 (C_12_H_8_O_2_, 1P), 12.9 ([C_7_H_4_O_3_]_4_, 2P). TGA: temperature range (weight loss): 70–230 °C (15.5%), 230–720 °C (28.6%) and 800–1000 °C (4.7%).

#### Synthesis of cyclotriphosphazene hexacarboxylate anion-intercalated LDH (LDH-PN-HC)

LDH-PN-HC was also prepared by a similar synthetic procedure as LDH-PN-DC using cyclotriphosphazene hexacarboxylic acid (L_3_) instead of cyclotriphosphazene dicarboxylic acid (L_1_). The matched molar ratio of Mg^2+^/Al^3+^/cyclotriphosphazene hexacarboxylic acid (L_3_) was 12 : 6 : 1 (18.80 mmol : 9.40 mmol : 1.57 mmol). Yield: 3.2 g. IR (ATR, cm^−1^): 3307(b), 1599(s), 1540(s), 1499(m), 1384(s), 1268(m), 1208(m), 1160(s), 1097(m), 1014(m), 970(s), 883(m), 789(m), 736(m), 665(s), 595(m), 558(m). Solid-state ^31^P NMR (162 MHz) *δ* (ppm): 12.7 ([C_7_H_4_O_3_]_6_, 3P). TGA: temperature range (weight loss): 70–230 °C (15.0%), 230–720 °C (29.0%) and 800–1000 °C (4.2%).

#### Preparation of the EP/LDH-PN composites

The EP/LDH-PN composites were prepared by a previously reported procedure with some modification.^44,45^ All three LDH-PN materials with different weight percentages (5 wt%, 10 wt%, 15 wt%, and 20 wt%) were dispersed in DMF using sonication for an hour. The desired amount of epoxy resin was added to the LDH-PN suspension and sonicated for an hour. Then, DMF was removed by a vacuum-assisted rota-evaporator and the resultant slurry was mixed at 120 °C for ten minutes. After reaching room temperature, the desired amount of curing agent (TETA) was added to the EP/LDH-PN mixture, which was mixed under sonication for 3 minutes. Likewise, the epoxy resin sample was prepared by the above procedure without the addition of LDH-PN.

#### Preparation of the EP/LDH-PN wood substrate and brush coating

To improve the adhesion, all the teak wood substrates were treated with electro-coated SiC grain emery paper of 220 grit size before the coating of the EP/LDH-PN composites. After mixing the curing agent, the EP/LDH-PN composites were immediately coated on the wood substrates using flat brushes. Then, the EP/LDH-PN wood substrates were cured at room temperature for 24 hours and post-cured at 80 °C overnight.

## Results and discussion

### Synthesis and characterization

**Scheme 1:**
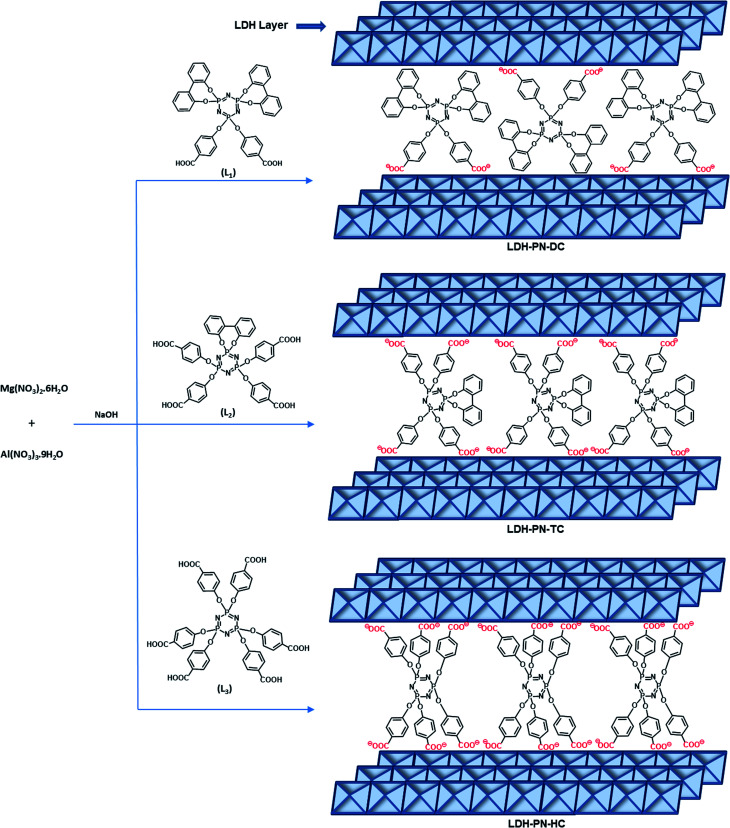
Synthesis of cyclotriphosphazene carboxylate anion-intercalated LDH.

Several layered inorganic solids have been used as host materials for the construction of inorganic–organic host–guest supramolecular structures by intercalating guest molecules or ions. The LDH-PN materials were synthesized by the homogeneous co-precipitation reaction between Mg(NO_3_)_2_ and Al(NO_3_)_3_, followed by the intercalation of different cyclotriphosphazene (PN) carboxylate anions (Scheme 1).^46^ For convenience, the PN-based dicarboxylic, tetracarboxylic and hexacarboxylic anion-intercalated LDHs were labelled LDH-PN-DC, LDH-PN-TC and LDH-PN-HC, respectively. The successful formation of the LDH-PN materials was confirmed by powder X-ray diffraction, FT-IR, thermogravimetric analysis, solid-state ^31^P NMR, nitrogen adsorption and desorption analysis (BET), high-resolution scanning electron microscopy and X-ray photoelectron spectroscopy.

**Fig. 1:**
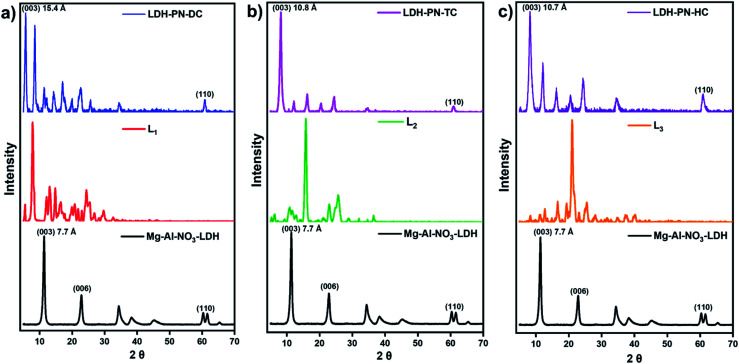
Powder XRD patterns of (a) Mg–Al–NO_3_-LDH, L_1_ and LDH-PN-DC; (b) Mg–Al–NO_3_-LDH, L_2_ and LDH-PN-TC; (c) Mg–Al–NO_3_-LDH, L_3_ and LDH-PN-HC.

The powder XRD patterns of Mg–Al–NO_3_-LDH, the three cyclotriphosphazene carboxylic acids (L_1_, L_2_, and L_3_) and the LDH-PN materials are shown in Fig. 1. The XRD patterns of the LDH-PN-DC, LDH-PN-TC and LDH-PN-HC materials confirm the formation of the LDH phase.^12,47,48^ Bragg peaks of each pattern at the lower angle region of 2*θ* < 25° emerge from the reflections of the basal plane passing through the metal sites in the hydroxide layers.^49^ Generally, the d_(003)_ peak is known as the basal spacing of each layer in LDH, which is defined as the total distance of the interlayer and thickness of the hydroxide layer (4.8 Å).^50,51^

Comparing Mg–Al–NO_3_-LDH with the LDH-PN materials, the basal *d*-spacings of LDH-PN-DC, LDH-PN-TC and LDH-PN-HC are shifted from 7.7 Å to 15.4 Å, 10.8 Å, and 10.7 Å, respectively. The increase in the *d*-spacing shows the successful intercalation of the cyclotriphosphazene carboxylate anions into the LDH layers, which makes the basal reflection of the acid-intercalated LDHs appear at a lower angle. In all the acid-intercalated LDHs, some of the carboxylic acid peaks are also present below the region of 2*θ* = 30°.

The last diffraction peak (*d*_(110)_) of 2θ ≃ 60.8° is responsible for the structural integrity of the LDH layers and was still maintained in all the acid-intercalated LDHs.^52^ In addition, the *d*_(110)_ spacing is related to the average distance between two metal ions in the hydroxide layers (*a*_M–M_ = 2*d*_(110)_), which is calculated to be 3.04 Å for the LDH-PN materials and is almost equal to the average distance between Mg–Al in Mg–Al–NO_3_-LDH (3.06 Å), with a metal fraction of 2. Thus, there is no significant change in the Mg–Al ratio, which indicates the complete precipitation of metal ions in the LDH-PN materials.^53^

**Fig. 2:**
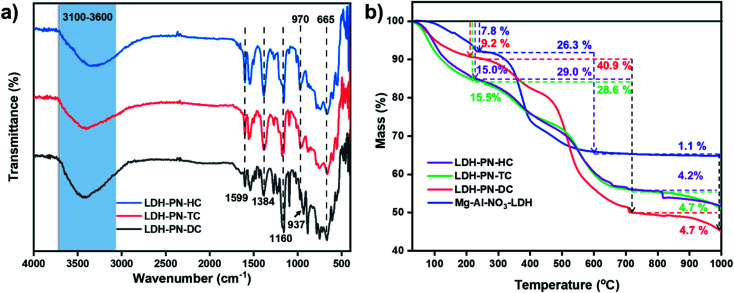
(a) FT-IR spectra of the LDH-PN materials and (b) thermogravimetric analysis of the LDH-PN and Mg–Al–NO_3_-LDH materials.

Fig. 2a shows the FT-IR spectra of the LDH-PN-DC, LDH-PN-TC and LDH-PN-HC materials. In the spectra of all the LDH-PN materials, the broad band in the range of 3100–3600 cm^−1^ is attributed to the hydroxyl group stretching vibrations in the LDH layers and water molecules due to the formation of hydrogen bonding between the interlayer water and hydroxyl groups of the host layers and the guest anions. The band around 665 cm^−1^ is assigned to the stretching vibration of M–O in the hydroxide layers.^53,54^ The characteristic band around 1160 cm^−1^ is ascribed to the P=N stretching vibration of the cyclotriphosphazene rings. The absorption bands around 970 cm^−1^ for LDH-PN-TC and LDH-PN-HC and 937 cm^−1^ for LDH-PN-DC are assigned to the P–O–C stretching vibration for the aromatic groups connected to the cyclic phosphorous atoms.^55^ The strong absorption bands around 1590 cm^−1^ and 1380 cm^−1^ are assigned to the asymmetric and symmetric stretching vibrations of the –COO^−^ groups, which shows that the carboxylic groups in the cyclotriphosphazenes are deprotonated to form their corresponding anions, indicating the successful intercalation of cyclotriphosphazene carboxylate anions into the LDH layers.^56^

The thermal stability, degradation behaviour and char formation ability of the LDH-PN and Mg–Al–NO_3_-LDH materials were studied by thermogravimetric analysis. As depicted in Fig. 2b, the whole thermal degradation process consists of three steps: the first step displays the loss of both physically adsorbed and interlayered water molecules around the temperature range of 70–230 °C.^57–59^ The percentages of weight loss for LDH-PN-DC, LDH-PN-TC, LDH-PN-HC and Mg–Al–NO_3_-LDH in the first step are about 9.2%, 15.5%, 15.0% and 7.8%, respectively. The second step involves simultaneous dehydroxylation in the LDH layers and the decomposition of the organic groups attached with cyclotriphosphazene in the intercalated acid anions around the temperature range of 230–720 °C.^60^ The percentages of weight loss in the second step for LDH-PN-DC, LDH-PN-TC, LDH-PN-HC and Mg–Al–NO_3_-LDH are about 40.9%, 28.6%, 29.0% and 26.3%, respectively. In addition, the percentages of weight loss in the first and second steps for LDH-PN-TC and LDH-PN-HC are almost the same but different from those of the LDH-PN-DC material. The final step shows the minimum weight loss of around <5% for the LDH-PN materials, and gives a higher char yield (45.2% for LDH-PN-DC, 51.2% for LDH-PN-TC and 51.8% for LDH-PN-HC) in the range of 800 to 1000 °C. It could be assumed that cross-linking reactions occur during pyrolysis, comprising the ring-opening polymerization reaction of the cyclotriphosphazene structure^34,61^ as well as the formation of a mixture of MgO and MgAl_2_O_4_ from the thermal degradation of the LDH layers as the final decomposition products.^62,63^

**Fig. 3:**
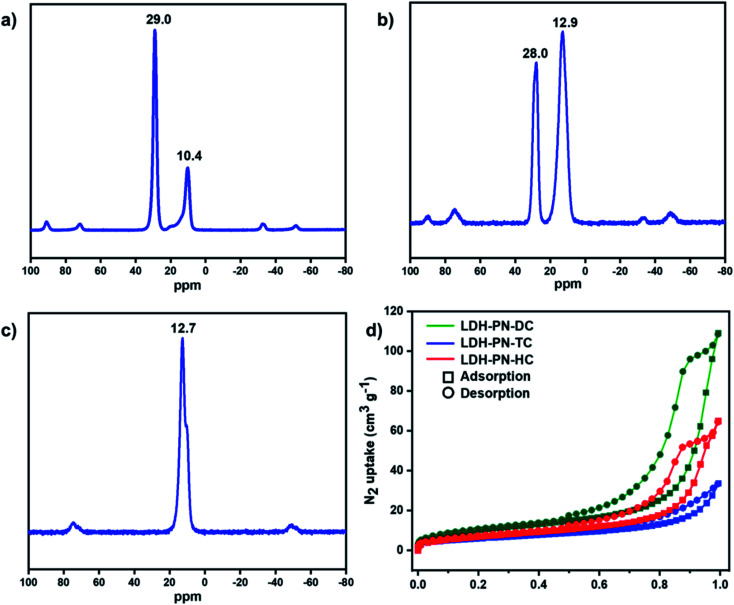
Solid-state ^31^P NMR spectra of (a) LDH-PN-DC, (b) LDH-PN-TC and (c) LDH-PN-HC; (d) N_2_ adsorption and desorption isotherms for LDH-PN-DC, LDH-PN-TC and LDH-PN-HC.

In the solution-state ^31^P NMR spectra of L_1_ and L_2_, two observed signals around 9.0 and 25.0 ppm are attributed to the phosphorous atoms in cyclotriphosphazene attached to the phenoxy groups and spiro groups, respectively (Fig. S7 and S15†).^64^ For the compound L_3_, only one singlet at 8.0 ppm is ascribed to the phosphorous atoms attached to the phenoxy groups, which indicates that all the phosphorous atoms in L_3_ remain magnetically equivalent.^43^ Similarly, the solid-state ^31^P NMR spectra of LDH-PN-DC and LDH-PN-TC display two signals (10.4 and 29.0 ppm for LDH-PN-DC; 12.9 and 28.0 ppm for LDH-PN-TC), and LDH-PN-HC shows only one signal at 12.7 ppm, as shown in Fig. 3a–c.^65^ Thus, the solid-state ^31^P NMR spectra of the LDH-PN materials are quite similar to those of their corresponding cyclotriphosphazene precursors, demonstrating that the cyclotriphosphazene carboxylate anions are intercalated successfully without any structural alteration to the LDH layers.

The N_2_ adsorption and desorption isotherms of LDH-PN-DC, LDH-PN-TC and LDH-PN-HC suggest that the LDH-PN materials display Type IV isotherms (Fig. 3d).^66^ The specific surface areas of LDH-PN-DC, LDH-PN-TC and LDH-PN-HC are 37.5 m^2^ g^−1^, 22.0 m^2^ g^−1^, and 25.6 m^2^ g^−1^, respectively. Compared to Mg–Al–NO_3_-LDH (74.6 m^2^ g^−1^) (Fig. S18†), the acid-intercalated LDHs possess a lower specific surface area due to the interlayer aggregation of three-dimensional cyclotriphosphazene carboxylate anions. These results indicate that the organic anions in cyclotriphosphazene can easily form dense structures with LDH.^47,51^

**Fig. 4:**
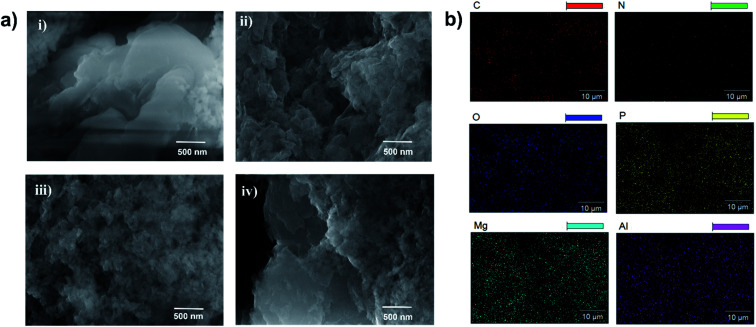
(a) HR-SEM images of (i) Mg–Al–NO_3_-LDH, (ii) LDH-PN-DC, (iii) LDH-PN-TC and (iv) LDH-PN-HC; (b) elemental mapping analysis of C, N, O, P, Mg, and Al in LDH-PN-DC.

To examine the surface morphology, the HR-SEM images of Mg–Al–NO_3_-LDH, LDH-PN-DC, LDH-PN-TC and LDH-PN-HC were captured and are shown in Fig. 4a. For Mg–Al–NO_3_-LDH, thin flat platelets were found with irregular edges and arranged in all space directions, forming some aggregates. All the three LDH-PN materials showed more aggregation with non-uniform/irregular morphology compared to Mg–Al–NO_3_-LDH. The guest anions are favourably stabilized on the basal face of the LDH structure during the LDH crystallization, causing the assembly with irregular morphology.^67^ Therefore, this kind of structural assembly will be conducive to inhibiting heat transfer and thus remarkably improving the thermal stability of the LDH-PN materials. The elemental composition and distribution of the LDH-PN materials by HR-SEM are shown in Fig. 4b and S19.† The specific elements of magnesium, aluminum, phosphorus and nitrogen are well distributed, indicating that the LDH-PN materials are fairly homogeneous. The uniform distribution of the four main elements is significant for their flame retardant properties.

**Fig. 5:**
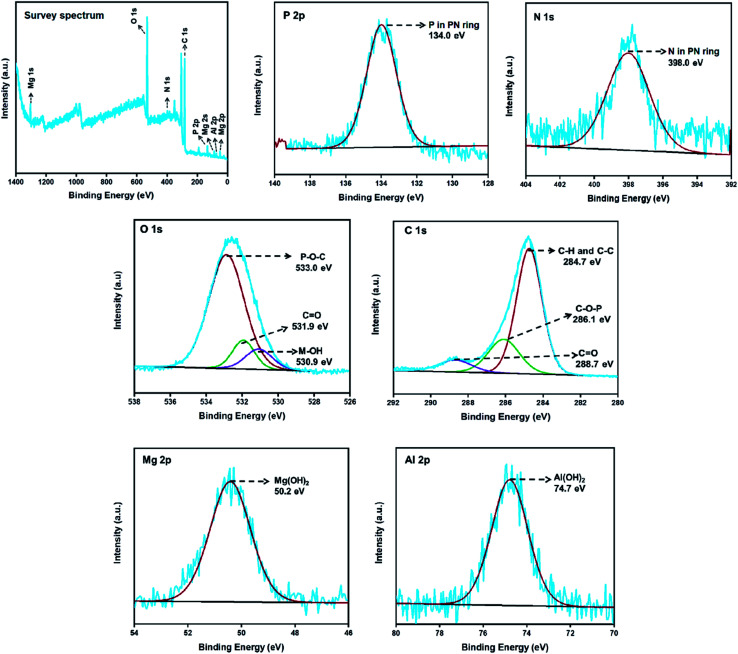
XPS survey spectrum; P 2p, N 1s, O 1s, C 1s, Mg 2p and Al 2p high-resolution spectra of LDH-PN-DC.

The elemental composition of the LDH-PN materials can be obtained from XPS analysis. The wide scan survey spectra and P 2p, N 1s, O 1s, Al 2p, and Mg 2p high-resolution spectra of LDH-PN-DC, LDH-PN-TC and LDH-PN-HC are presented in Fig. 5, S20 and S21,† respectively. The spectra for P 2p and N 1s of the LDH-PN materials reveal two major peaks at 134.0 ± 0.2 eV and 398.0 ± 0.1 eV, respectively, identifying the presence of P and N in the cyclotriphosphazene ring.^68–73^ The spectra for O 1s of LDH-PN are divided into three peaks that indicate the presence of three different oxygen environments in the LDH-PN materials. The first peak at about 533.0 ± 0.4 eV is ascribed to the P–O–C bond in the intercalated anions, the second peak at 531.9 ± 0.1 eV is assigned to the C=O present in the carboxylate groups, and the final peak at about 530.7 ± 0.2 eV is attributed to the hydroxyl groups present in the hydroxide layers. Similarly, the spectra for C 1s of the LDH-PN materials are distributed into three peaks that arise from the intercalated aromatic carboxylate anions. The peaks at 284.7 ± 0.3 eV, 286.1 ± 0.6 eV and 288.7 ± 0.2 eV are attributed to the C–C and C–H in the aromatic species, C–O–P in the PN species, and C=O in the carboxylate anions, respectively.^74,75^ The peaks at 74.7 ± 0.1 eV for Al 2p and 50.2 ± 0 eV for Mg 2p are assigned to Al(OH)_2_ and Mg(OH)_2_, respectively, in the form of LDH layers in the LDH-PN materials.^76,77^ These results indicate the successful intercalation of cyclotriphosphazene carboxylate anions into the LDH layers.

### Flame retardant properties

Wood is commonly used for furniture and interior decoration materials owing to its abundance and outstanding mechanical properties. The flammable nature of wood is the main constraint on its use in interior environments, particularly in heavily populated zones. There is a high demand for state-of-the-art flame retardant technologies to protect wood from fire by coating it with highly flammable epoxy resin (EP).^45,78,79^ Here, the EP/LDH-PN composites coated on the wood substrates exhibited flame retardancy to epoxy resin as well as protected the wood from flame. The assessment of flammability was carried out using a vertical burning test (UL-94 V) and the limiting oxygen index (LOI) test. The standard sizes of the wood substrates used for the UL 94 V test and LOI test were 130 × 13 × 5 mm^3^ (ASTM D3801) and 150 × 7 × 3 mm^3^ (ASTM D2863), respectively. In addition, the thickness of the epoxy resin and EP/LDH-PN composite-coated wood substrate was computed from cross-sectional microscopic images by measuring the thickness at 100 different points, and the value was 346.13 ± 16 μm (Fig. S22†). The epoxy resin-coated wood substrate and three EP/LDH-PN composite-coated wood substrates with different weight percentages of LDH-PN materials from 5 to 20 wt% were used in the vertical burning test (UL-94 V). For example, EP/LDH-PN-DC-20 wt% denotes that the composite contains 20 wt% LDH-PN-DC.

**Fig. 6:**
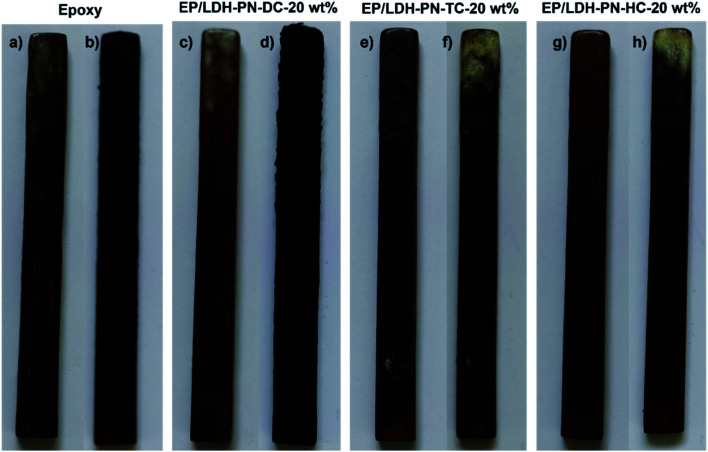
Digital photos before (a, c, e and g) and after (b, d, f and h) the UL-94 vertical burning tests of the epoxy and EP/LDH-PN composite-coated wood substrates.

**Fig. 7:**
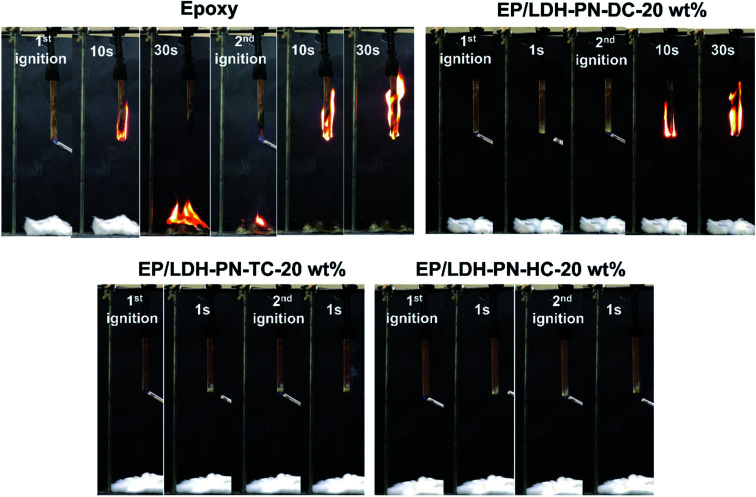
Digital photos of the pure epoxy resin, EP/LDH-PN-DC-20 wt%, EP/LDH-PN-TC-20 wt%, and EP/LDH-PN-HC-20 wt% composite-coated wood substrates during the UL-94 V vertical burning process.

The pure epoxy resin-coated wood substrate did not show any flame retardant properties in the UL-94 V tests. The entire length of the epoxy-coated wood substrate was completely burned after the second ignition time (30 seconds) in the UL-94 V test. The EP/LDH-PN-DC-20 wt% wood substrate did not capture the flame after the first ignition time, but during the second ignition time, it captured the flame and was completely burned after 30 seconds. Therefore, the EP/LDH-PN-DC-20 wt% wood substrates also failed the UL-94 V test (Fig. 6 and 7). However, EP/LDH-PN-TC-20 wt% and EP/LDH-PN-HC-20 wt% showed non-flammable behaviour and were classified with a *V*_0_ rating in the vertical burning test (Fig. 6 and 7). Both samples did not capture the flame during the first and the second ignition times, which proves the importance of the nature of cyclotriphosphazene carboxylate anions within the LDH layers. The cyclotriphosphazene dicarboxylate anions (L_1_) in LDH-PN-DC, tetracarboxylate anions (L_2_) in LDH-PN-TC and hexacarboxylate anions (L_3_) in LDH-PN-HC consist of two spiro groups with two carboxylate binding sites, one spiro group with four carboxylate binding sites and only six carboxylate binding sites, respectively. Thus, the smaller number of spiro groups and more binding sites assist the effective dispersion of epoxy resin in the LDH layers, resulting in the superior flame retardant properties of the EP/LDH-PN-TC-20 wt% and EP/LDH-PN-HC-20 wt% composites. In other words, the more spiro groups and smaller number of binding sites inhibit the dispersion of epoxy resin in the LDH layers, leading to the separate aggregation of LDH-PN-DC in the EP/LDH-PN-DC-20 wt% composite, which failed in terms of flame retardant properties. Furthermore, the flame retardant properties of the EP/Mg–Al–NO_3_-LDH-20 wt%, EP/L_1_-20 wt% and EP/L_3_-20 wt%-coated wooden samples were tested in the UL-94 V test and all these samples failed by demonstrating their highly flammable behaviour (Fig. S23†). Hence, compared to EP/Mg–Al–NO_3_-LDH, EP/L_1_ and EP/L_3_, the flame retardant properties of the EP/LDH-PN composites are improved due to the synergistic flame retardant effect of the combination of LDH and cyclotriphosphazene in the epoxy resins.

The limiting oxygen index (LOI) is an important parameter for evaluating the flame retardancy of samples, which measures the minimum oxygen concentration of a flowing gas comprising oxygen and nitrogen required to support downward flame combustion. Epoxy coated on the wood substrate was highly flammable and had an LOI value of 23.2 vol%. However, EP/LDH-PN-DC coated on the wood substrate showed a lower LOI value of 22.1 vol% than the epoxy wood substrate. Compared with those of the epoxy and EP/LDH-PN-DC-coated wood substrates, the LOI values of the EP/LDH-PN-TC and EP/LDH-PN-HC-coated wood substrates increased to 27.7 vol% and 29 vol%, respectively. Therefore, the result of the LOI test indicates that both the EP/LDH-PN-TC and EP/LDH-PN-HC composites impart excellent flame retardancy to epoxy resin on wood substrates, which demonstrates the significant role of the spiro groups and binding sites present in cyclotriphosphazene.

**Scheme 2:**
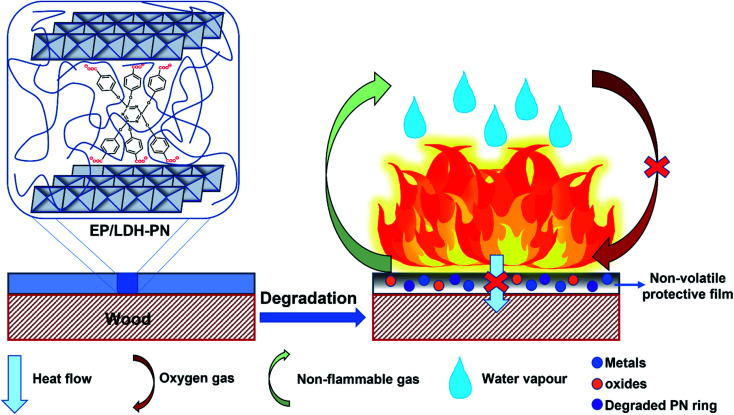
Schematic illustration of the flaming of the EP/LDH-PN composite-coated wood substrate.

Based on the above analysis, we proposed a mechanism for the flame retardancy of the EP/LDH-PN composites on the wood substrate, as shown in Scheme 2.

During the combustion process, the decomposition of cyclotriphosphazene and the LDH layers produces degraded PN rings with the emission of non-flammable gases (CO_2_ and N_2_) and metal oxides with water vapour, respectively.^32,80,81^ This synergistic effect of cyclotriphosphazene and LDH in the EP/LDH-PN composite leads to the formation of a white layer of non-volatile protective film on the surface of the wood substrate. Thus, the non-volatile protective film insulates the wood substrate from the air, absorbs the heat, and increases the ignition time, which prevents oxygen supply during the combustion process.

## Conclusion

A series of environmentally friendly LDHs have been prepared with different cyclotriphosphazene carboxylate anions as intercalating guest molecules and their flame retardant properties have been examined. Their powder XRD patterns demonstrate that the interlayer distance of all the LDH-PN materials increases upon the intercalation reactions of LDH with cyclotriphosphazene carboxylate anions compared with that of pristine Mg–Al–NO_3_-LDH, and the diffraction peak at 2θ ≃ 60.8° in the LDH-PN materials confirms the formation of the layered structures. From TGA, the first and second weight loss percentages for LDH-PN-TC and LDH-PN-HC are found to be almost the same but different from that of the LDH-PN-DC material due to the number of binding sites of the cyclotriphosphazene carboxylate anions. Fourier transform infrared spectroscopy and X-ray photoelectron spectroscopy further confirmed the presence of cyclotriphosphazene carboxylate anions in the LDH layers. Flame retardant tests (UL-94 V and LOI tests) were performed using epoxy resin with varying weight percentages of LDH-PN materials (5–20 wt%) coated on wood substrates. EP/LDH-PN-TC-20 wt% and EP/LDH-PN-HC-20 wt% passed the UL-94 V test with a *V*_0_ rating and showed higher LOI values, which demonstrated that these materials do not show burning properties and completely protect the wood substrate from the flame. However, the EP/LDH-PN-DC samples with different ratios of LDH-PN-DC from 5% to 20 wt% showed no self-extinguishing properties and burned to completion in the UL-94 test and showed a lower LOI value of 22.1 vol%. Hence, the incorporation of either LDH-PN-HC or LDH-PN-TC into the epoxy resin significantly improves the flame retardant properties compared to that in LDH-PN-DC because of their cyclotriphosphazene structure with more binding carboxylate sites and less or no bulky spiro groups that result in the good dispersion of LDH-PN-HC or LDH-PN-TC in the polymer matrix, and the unique combination of LDH and cyclotriphosphazene resulted in a synergistic flame retardant effect. Therefore, this work provides a feasible strategy to design sustainable halogen-free flame retardant materials to resolve the common issues related to environmental safety and human health.

## Author contributions

Conceived and designed the experiments: V. J. and S. S.; performed the experiments: V. J.; measured and analyzed the spectroscopic data: V. J. and S. S.; wrote the paper: S. S. and V. J.

## Conflicts of interest

There are no conflicts to declare.

## Supplementary Material

RA-012-D2RA02586H-s001
