# Supplementary material for: Halogen-free layered double hydroxide-cyclotriphosphazene carboxylate flame retardants: effects of cyclotriphosphazene di, tetra and hexacarboxylate intercalation on layered double hydroxides against the combustible epoxy resin coated on wood substrates
Source: RSC Adv. 2022 Aug 16;12(36):23322–36. doi: 10.1039/d2ra02586h (PMC9380775; doi:10.1039/d2ra02586h)
Supplement: RA-012-D2RA02586H-s001 [file RA-012-D2RA02586H-s001.pdf]

Electronic Supplementary Material (ESI) for RSC Advances.
This journal is © The Royal Society of Chemistry 2022

Electronic Supplementary Information

Halogen-free layered double hydroxides-cyclotriphosphazene carboxylates flame retardants: Effects of cyclotriphosphazene di, tetra and hexa carboxylates intercalation on layered double hydroxides against the combustible epoxy resin coated on wood substrate

Velusamy Jeevananthan, Swaminathan Shanmugan*

Department of Chemistry, Faculty of Engineering and Technology, SRM Institute of Science and Technology, Kattankulathur-603203, Tamil Nadu, India.

Corresponding author: Swaminathan Shanmugan (shanmugs2@srmist.edu.in, shanmugan0408@gmail.com)

Contents

Synthesis of Cyclotriphosphazene dicarboxylic acid [*dispiro*-$N_3P_3(O_2C_{12}H_8)_2$ $(OC_6H_4COOH)_2$] (**L_1**)

Synthesis of Cyclotriphosphazene Tetracarboxylic acid [*spiro*-$N_3P_3(O_2C_{12}H_8)$ $(OC_6H_4COOH)_4$](**L_2**)

Fig. S1 ^{1}H-NMR spectrum of compound **2**.

Fig. S2 ^{13}C-NMR spectrum of compound **2**.

Fig. S3 ^{31}P-NMR spectrum of compound **2**.

Fig. S4 ESI-MS of compound **2**.

Fig. S5 ^{1}H-NMR spectrum of compound **L_1**.

Fig. S6 ^{13}C-NMR spectrum of compound **L_1**.

Fig. S7 ^{31}P-NMR spectrum of compound **L_1**.

Fig. S8 ESI-MS of compound **L_1**.

Fig. S9 ^{1}H-NMR spectrum of compound **4**.

Fig. S10 ^{13}C-NMR spectrum of compound **4**.

Fig. S11 ^{31}P-NMR spectrum of compound **4**.

Fig. S12 ESI-MS of compound **4**.

Fig. S13 ^{1}H-NMR spectrum of compound **L_2**.

Fig. S14 ^{13}C-NMR spectrum of compound **L_2**.

Fig. S15 ^{31}P-NMR spectrum of compound **L_2**.

Fig. S16 ESI-MS of compound **L_2**.

Fig. S17 FT-IR spectra of compounds **2**, **L_1**, **4** and **L_2**.

Fig. S18 N_2 adsorption and desorption isotherm for Mg-Al-NO_3-LDH

Fig. S19 Elemental mapping analysis of C, N, O, Mg, Al, P of a) LDH-PN-TC, b) LDH-PN-HC

Fig. S20 (a) XPS survey spectrum of LDH-PN-TC; (b)) P 2p, N 1s, O 1s, C 1s, Al 2p and Mg 2p High-resolution spectra of LDH-PN-TC

Fig. S21 (a) XPS survey spectrum of LDH-PN-HC; (b) P 2p, N 1s, O 1s, C 1s, Al 2p and Mg 2p High-resolution spectra of LDH-PN-HC.

Fig. S22 (a) A microscopic image of the wood and EP/LDH-PN composite coated wood substrate and the inset shows a cross-sectional image of EP/LDH-PN composite coated wood substrate; (b) Histogram diagram for the calculation of average thickness of the EP/LDH-PN composite.

Fig. S23. Digital photos of EP/L_1-20 wt%, EP/L_3-20 wt%, EP/Mg-Al-NO_3-LDH-20 wt% composite coated wood substrates during the UL94 vertical burning process.

Synthesis of Cyclotriphosphazene dicarboxylic acid [*dispiro*-$N_3P_3(O_2C_{12}H_8)_2$ $(OC_6H_4COOH)_2$](L_1)

In a double neck 250ml round-bottom flask equipped with a condenser, methyl 4-hydroxy benzoate (26.82mmol), *dispiro*-$N_3P_3(O_2C_{12}H_8)Cl_2$ **(1)** (12.19mmol), potassium carbonate (36.57mmol) and acetone (160ml) were added. The mixture was reflux under nitrogen atmosphere for 24 h, and then cooled to room temperature. After the solvent was removed by rota-evaporator, the crude product was extracted twice with dichloromethane (2 × 80 mL). The solvent was evaporated under vacuum to give the light yellowish gel. It is immersed in hexane for overnight to give *dispiro*-$N_3P_3(O_2C_{12}H_8)_2(OC_6H_4COOCH_3)_2$ **(2)** as a pale yellow solid. Yield: 90%. ^1H NMR (500 MHz, $CDCl_3$) δ (ppm): 8.10 (d, J = 8.6 Hz, 4H), 7.51 (dd, J = 7.6, 1.6 Hz, 4H), 7.43 (dd, J = 8.7, 1.2 Hz, 4H), 7.38 (t, J = 8.5 Hz, 4H), 7.31 (t, J = 7.4 Hz, 4H), 7.07 (d, J = 8.0 Hz, 4H), 3.92 (s, 6H). ^{13}C NMR (126 MHz, $CDCl_3$) δ (ppm): 166.40, 154.37, 147.94, 131.48, 129.70, 128.66, 127.25, 126.24, 121.75, 121.06, 52.27. ^{31}P NMR (203 MHz, $CDCl_3$) δ (ppm): 25.11 (d, J = 94.0 Hz, $C_{12}H_8O_2$, 2P), 8.83 (t, J = 94.1 Hz, $(C_8H_7O_3)_2$, 1P). Mass spectrum (ESI = 70 eV): m/z = 806.2000 $[M]^+$. FT-IR (ATR, cm^{-1}): 1712(s), 1603(s), 1503(s), 1476(m), 1435(s), 1273(s), 1226(s), 1166(s), 1090(s), 1035(m), 1012(s), 968(s), 936(s), 882(s), 774(s), 754(s), 719(s), 690(s), 650(m), 638(m), 603(s), 557(s).

A quantity of **(2)** (11.17mmol) was added into the solution of sodium hydroxide (111.73mmol in 300ml of methanol). The mixture was reflux at 80^0C for 24 h. After the evaporation of the solvent, the obtaining solid was dissolved in water and acidified with diluted hydrochloric acid (pH=2~3) with stirring to give the white slurry as the product **(L_1)**. The resulting slurry was centrifuged and washed with water (until pH=7) dried at 80^0C in a hot air oven for overnight. Yield: 82%.^1H NMR (500 MHz, DMSO) δ (ppm): 13.13 (s, 2H),

8.13 (d, J = 8.5 Hz, 4H), 7.68 (d, J = 7.5 Hz, 4H), 7.49 (ddd, J = 19.7, 15.1, 7.4 Hz, 12H), 7.21 (d, J = 8.0 Hz, 4H). ^{13}C NMR (126 MHz, DMSO) δ (ppm): 167.05, 153.61, 147.49, 132.10, 130.80, 130.42, 128.81, 128.23, 127.29, 122.03, 121.46. ^{31}P NMR (203 MHz, DMSO) δ (ppm):24.98 (d, J = 93.5 Hz, $C_{12}H_8O_2$, 2P), 9.38 (t, J = 93.5 Hz, $(C_7H_5O_3)_2$, 1P). Mass spectrum (ESI = 70 eV): m/z = 778.0500 $[M+1]^+$. FT-IR (ATR, cm^{-1}): 1693(s), 1600(s), 1501(s), 1475(s), 1432(s), 1274(s), 1228(m), 1160(s), 1093(s), 1011(m), 971(m), 932(s), 883(s), 777(s), 751(s), 715(s), 609(s), 537(s).

Synthesis of Cyclotriphosphazene Tetracarboxylic acid [*spiro*-$N_3P_3(O_2C_{12}H_8)$ $(OC_6H_4COOH)_4$](L_2)

Compound **(L_2)** was synthesized by the similar synthetic procedure of Compound (**L_1**) using *spiro*-$N_3P_3(O_2C_{12}H_8)Cl_4$ **(3)** instead of compound **(1)**.

***spiro*-$N_3P_3(O_2C_{12}H_8)(OC_6H_4COOCH_3)_4$ (4):** Yield: 79%. ^{1}H NMR (500 MHz, $CDCl_3$) δ (ppm): 7.97 (d, J = 8.8 Hz, 8H), 7.52 (d, J = 7.2 Hz, 2H), 7.38 – 7.31 (m, 4H), 7.19 (d, J = 8.7 Hz, 8H), 6.79 (dd, J = 9.1, 1.6 Hz, 2H), 3.92 (s, 12H). ^{13}C NMR (126 MHz, $CDCl_3$) δ (ppm): 166.04, 153.91, 147.68, 131.38, 129.84, 128.51, 127.32, 126.37, 121.52, 120.83, 52.25. ^{31}P NMR (203 MHz, $CDCl_3$) δ (ppm): 24.35 (t, J = 94.5 Hz, $C_{12}H_8O_2$, 1P), 8.29 (d, J = 94.4 Hz, $(C_8H_7O_3)_4$, 2P). Mass spectrum (ESI = 70 eV): m/z = 925.0000 $[M+1]^+$. FT-IR (ATR, cm^{-1}): 1712(s), 1600(s), 1502(s), 1479(m), 1272(s), 1233(s), 1181(s), 1161(s), 1095(s), 1013(s), 944(s), 895(s), 853(s), 767(s), 751(s), 718(s), 689(s), 630(s), 610(s), 557(s).

***spiro*-$N_3P_3(O_2C_{12}H_8)(OC_6H_4COOH)_4$ (L_2):** Yield: 81%. ^{1}H NMR (500 MHz, DMSO) δ (ppm): 13.14 (s, 4H), 7.99 (d, J = 8.7 Hz, 8H), 7.65 (dd, J = 5.8, 3.7 Hz, 2H), 7.43 (dd, J = 5.8, 3.6 Hz, 4H), 7.23 (d, J = 8.7 Hz, 8H), 6.76 (dd, J = 7.1, 3.7 Hz, 2H). ^{13}C NMR (126 MHz, DMSO) δ (ppm): 166.86, 153.39, 147.26, 131.87, 130.61, 130.43, 128.58, 128.05, 127.35, 121.35. ^{31}P NMR (203 MHz, DMSO) δ (ppm): 24.60 (t, J = 93.2 Hz $C_{12}H_8O_2$, 1P),

8.84 (d, J = 93.2 Hz, $(C_7H_5O_3)_4$, 2P). Mass spectrum (ESI = 70 eV): m/z = 866.0550 $[M-1]^+$. FT-IR (ATR, cm^{-1}): 1683(s), 1597(s), 1503(s), 1474(m), 1421(s), 1315(s), 1271(s), 1177(s), 1153(s), 1097(s), 1015(m), 945(s), 880(s), 854(s), 804(s), 771(s), 754(s), 736(s), 715(s), 689(s), 625(s), 613(s), 545(s).

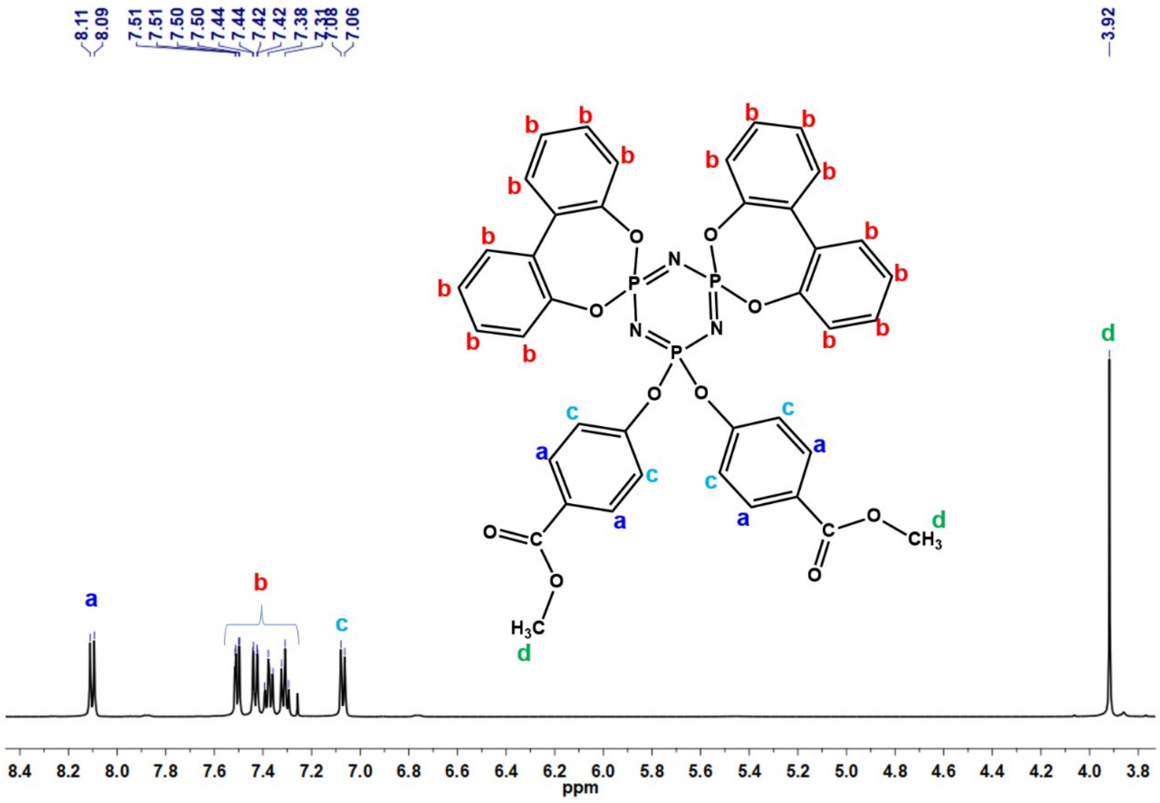

Fig. S1 ^{1}H-NMR spectrum of compound **2**.

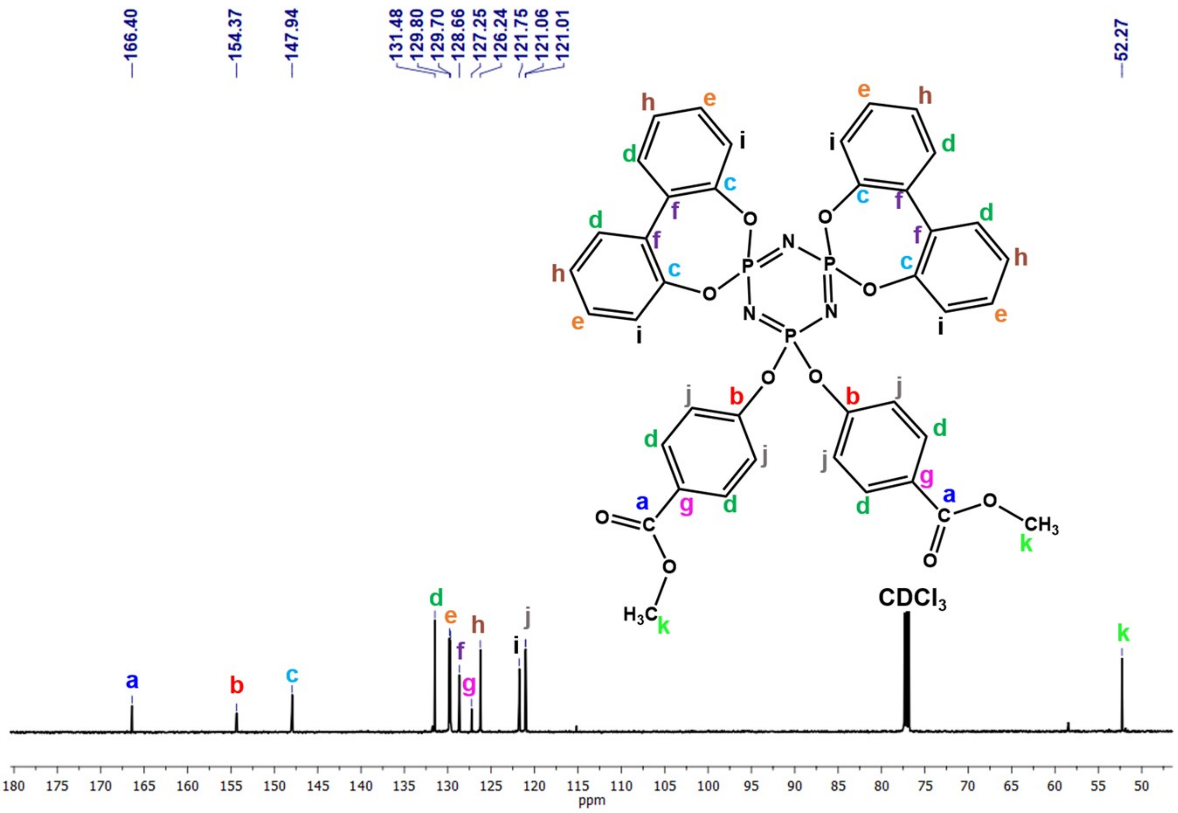

Fig. S2 ^{13}C-NMR spectrum of compound **2**.

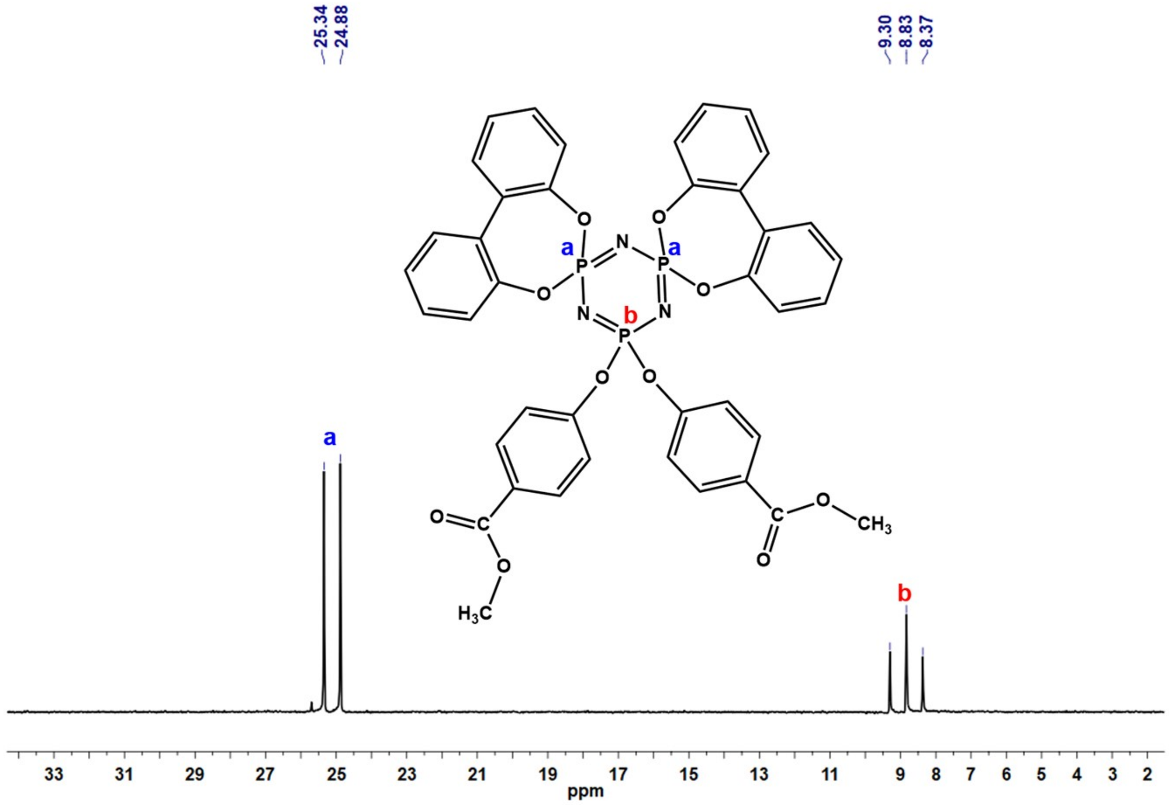

Fig. S3 ^{31}P-NMR spectrum of compound **2**.

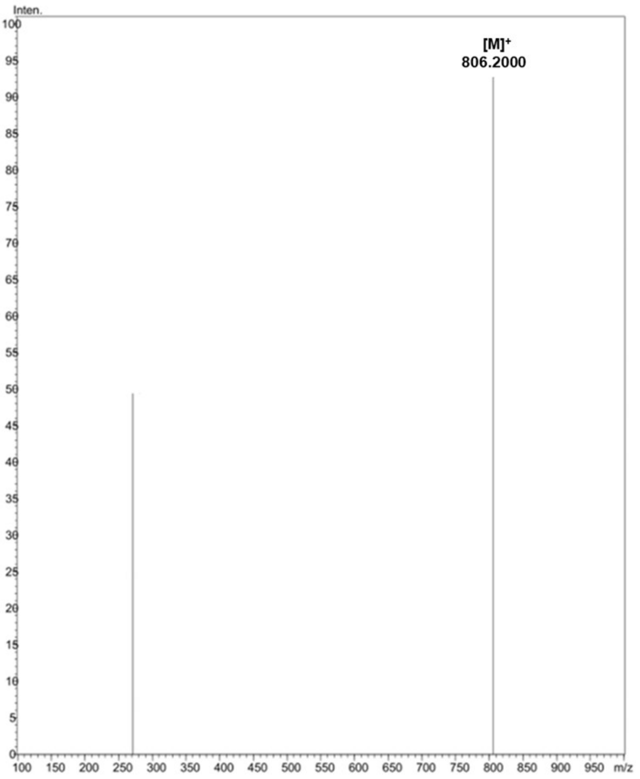

Fig. S4 ESI-MS of compound **2**.

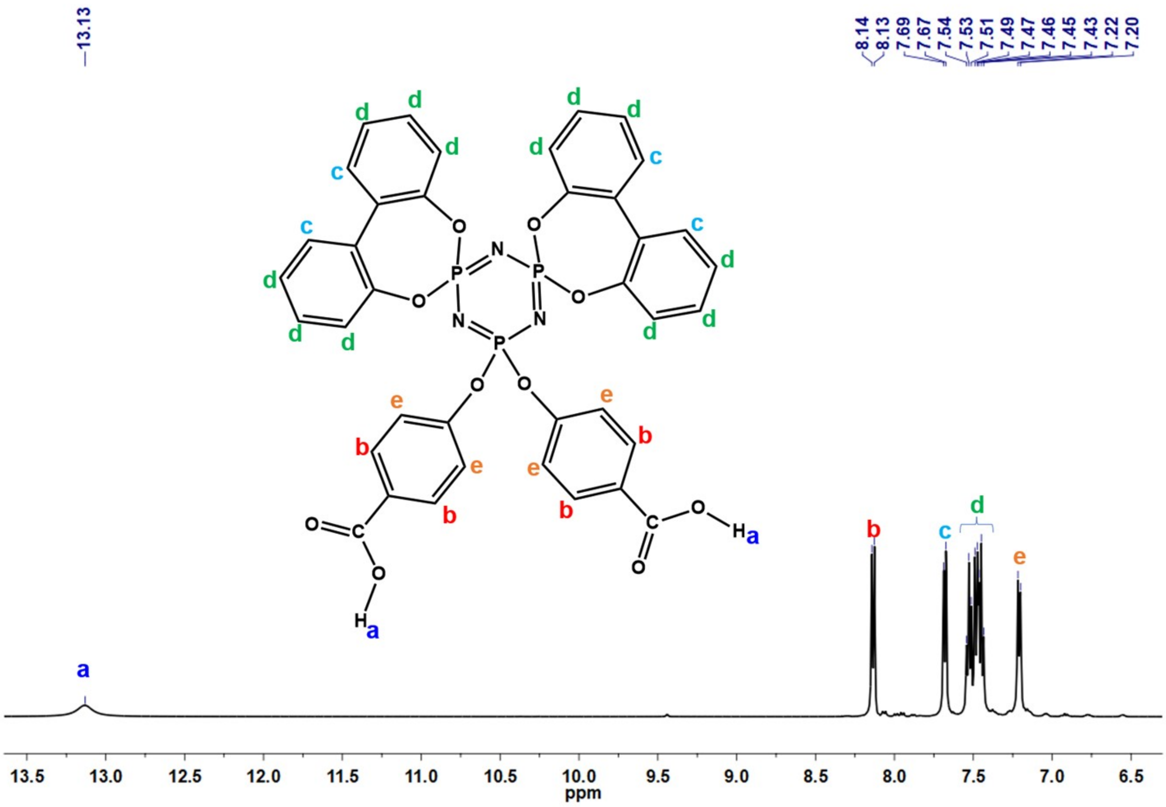

Fig. S5 ^{1}H-NMR spectrum of compound $\mathbf{L_1}$.

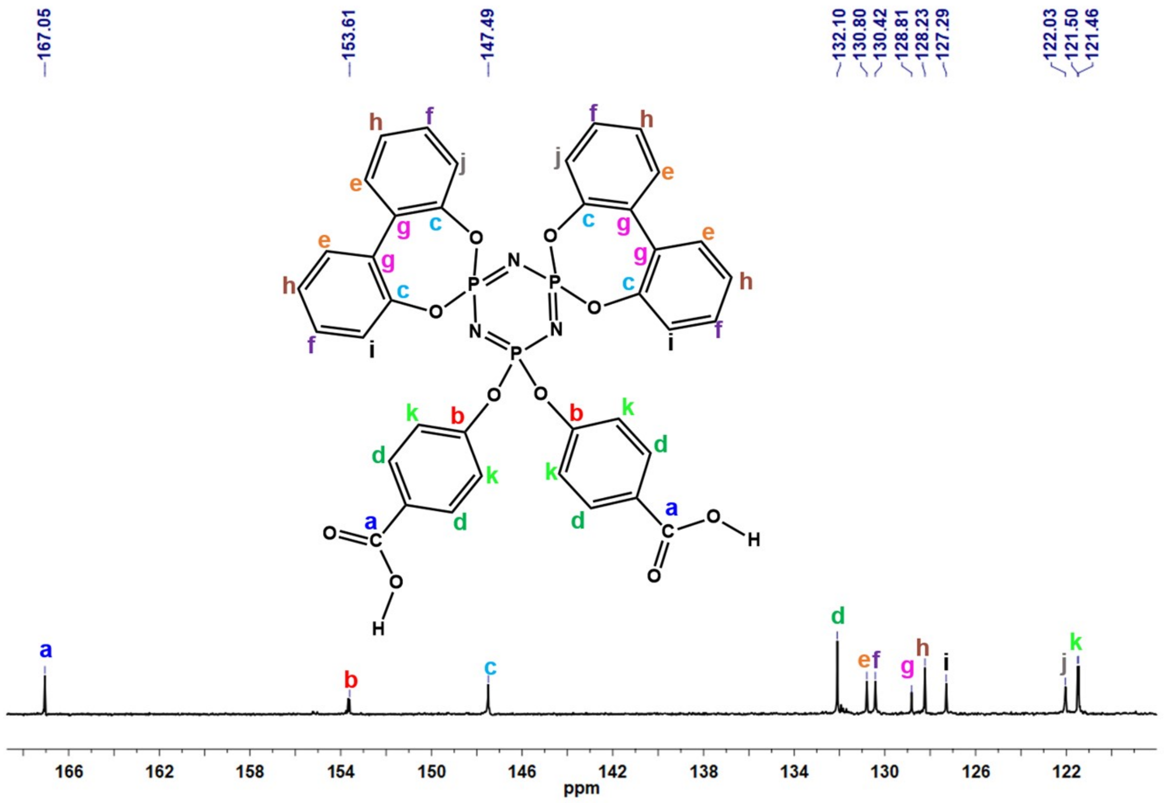

Fig. S6 ^{13}C-NMR spectrum of compound $\mathbf{L_1}$.

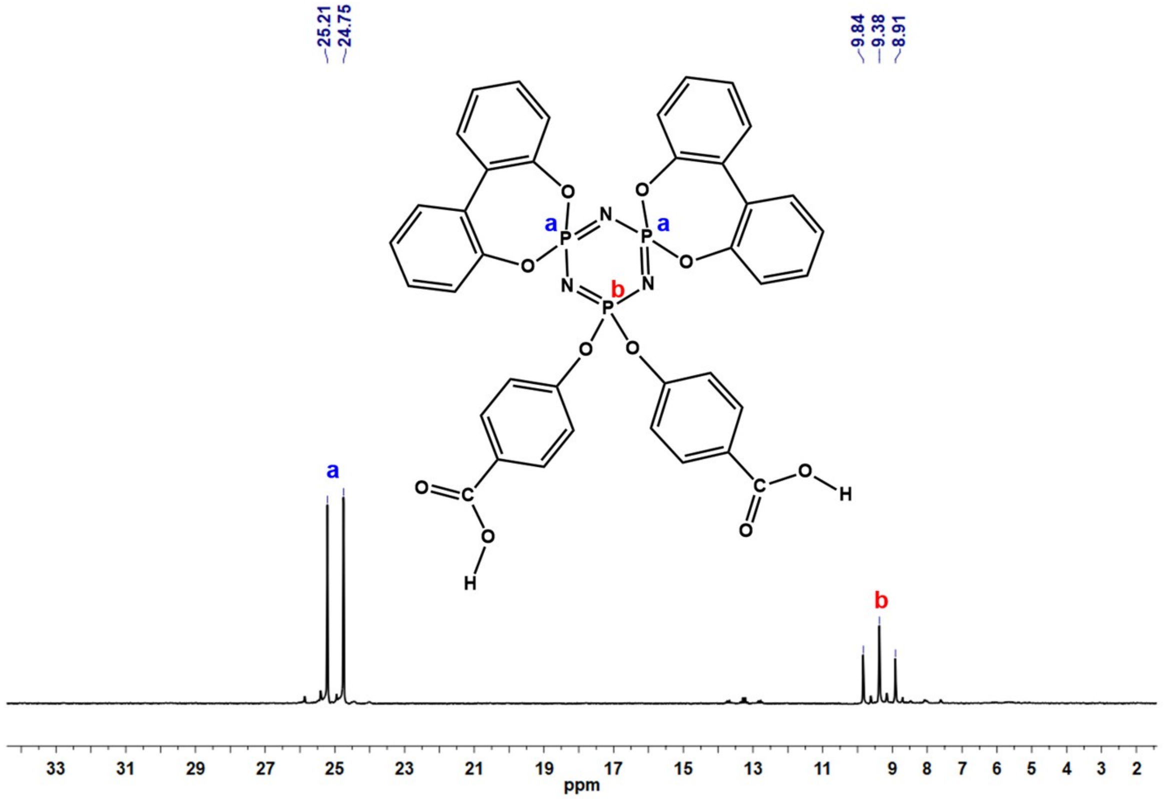

Fig. S7 ^{31}P-NMR spectrum of compound $\mathbf{L_1}$.

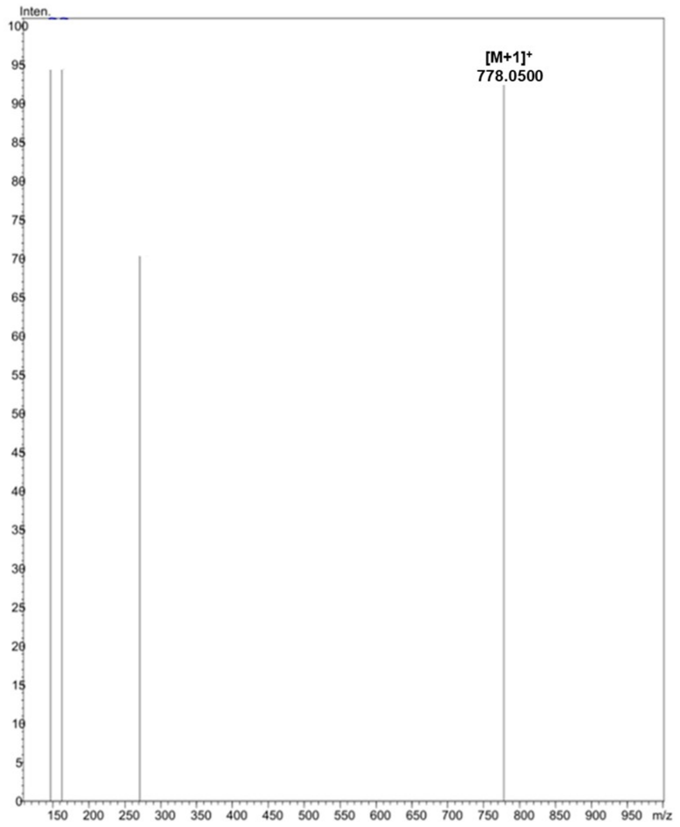

Fig. S8 ESI-MS of compound $\mathbf{L_1}$

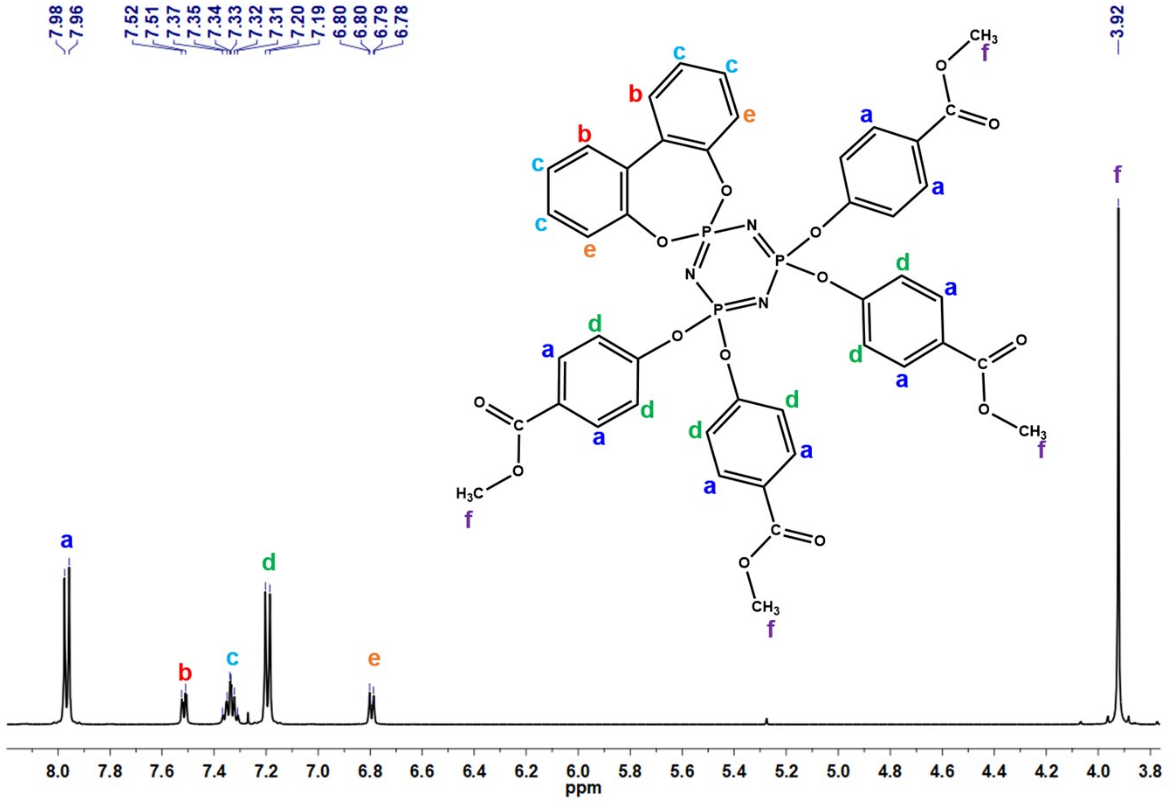

Fig. S9 ^{1}H-NMR spectrum of compound **4**.

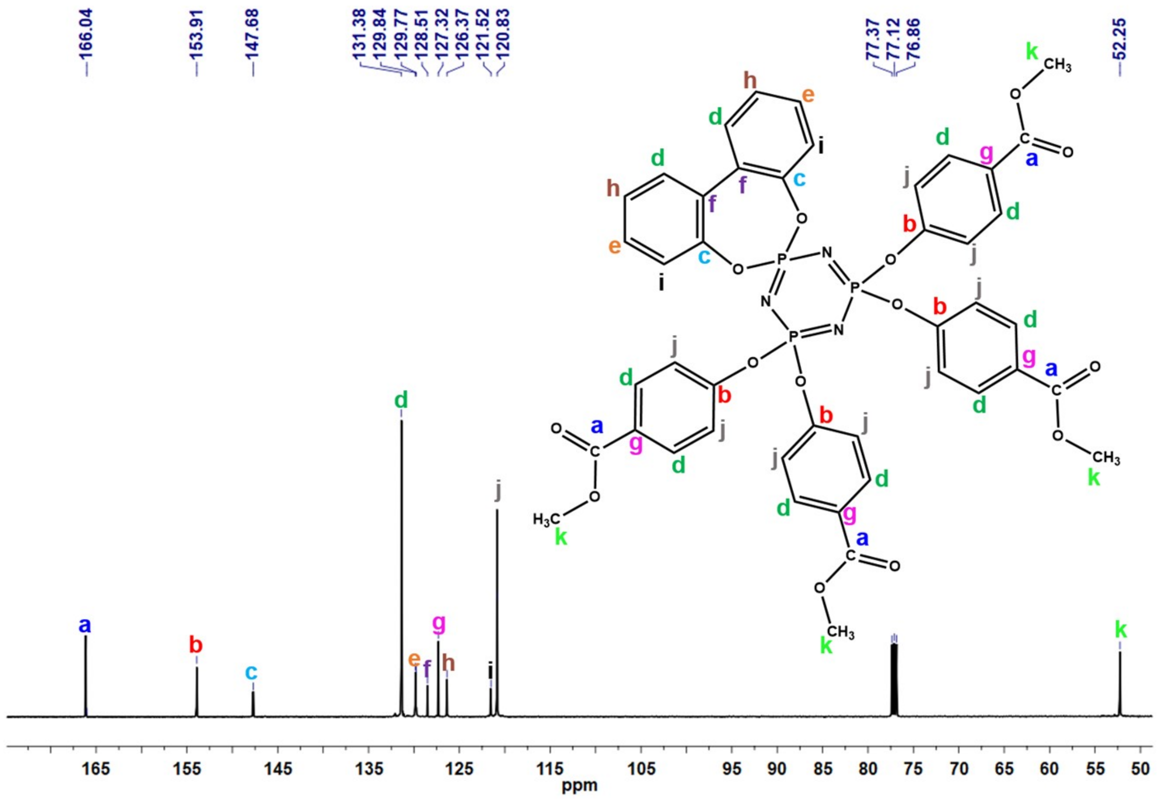

Fig. S10 ^{13}C-NMR spectrum of compound **4**.

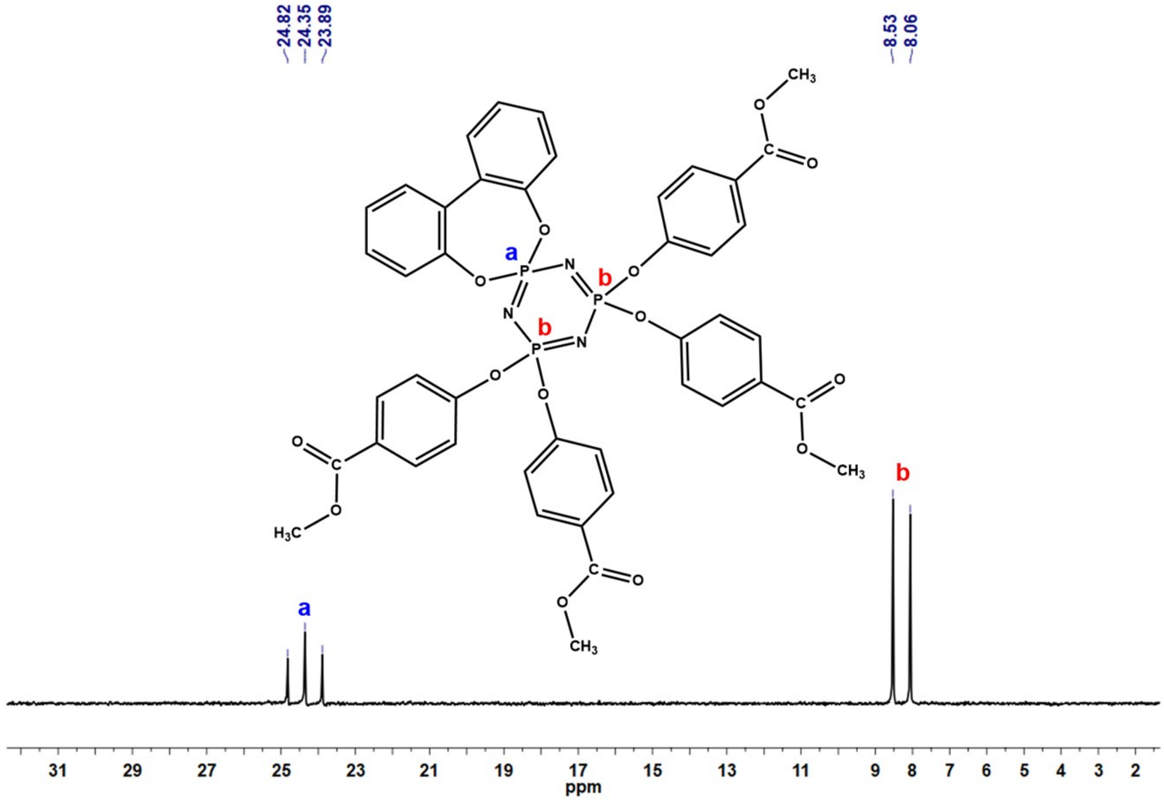

Fig. S11 ^{31}P-NMR spectrum of compound **4**.

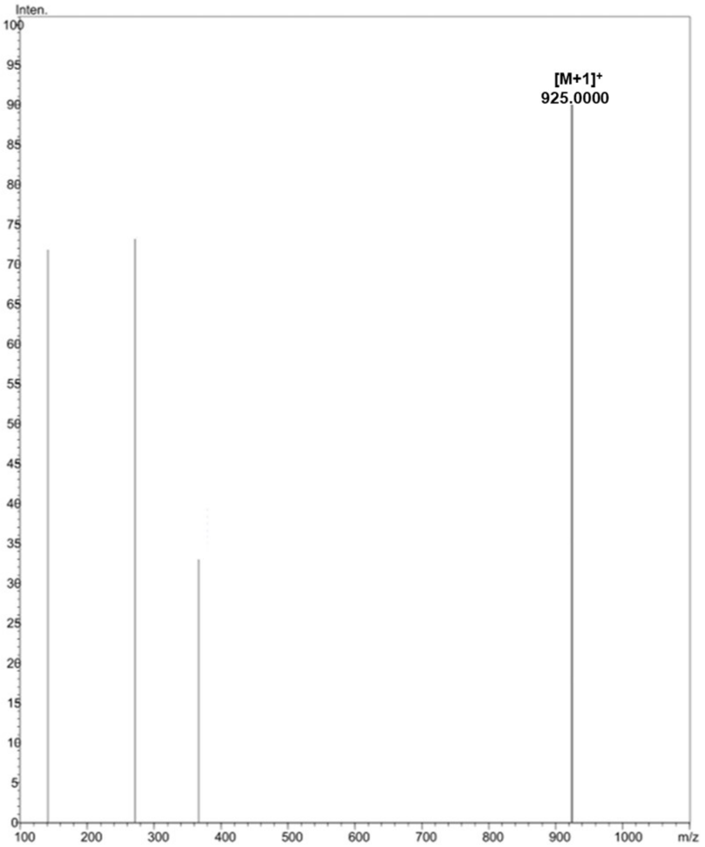

Fig. S12 ESI-MS of compound **4**.

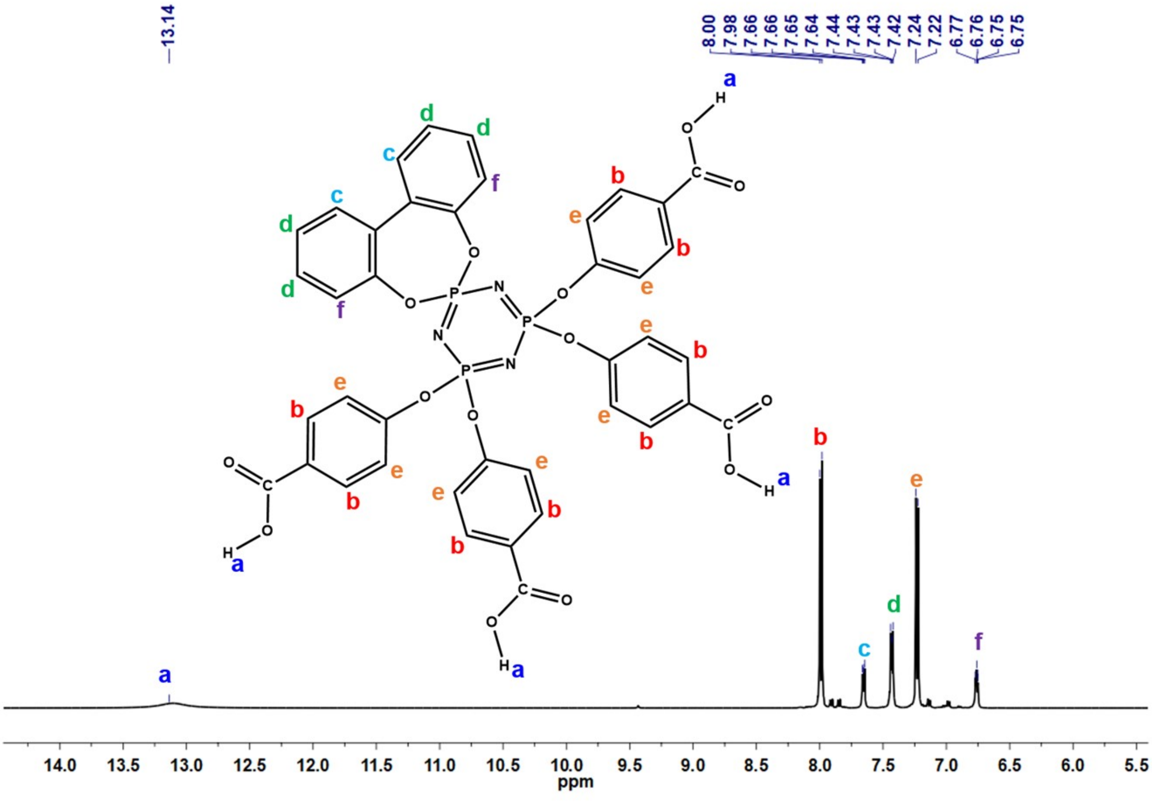

Fig. S13 ^{1}H-NMR spectrum of compound $\mathbf{L_2}$.

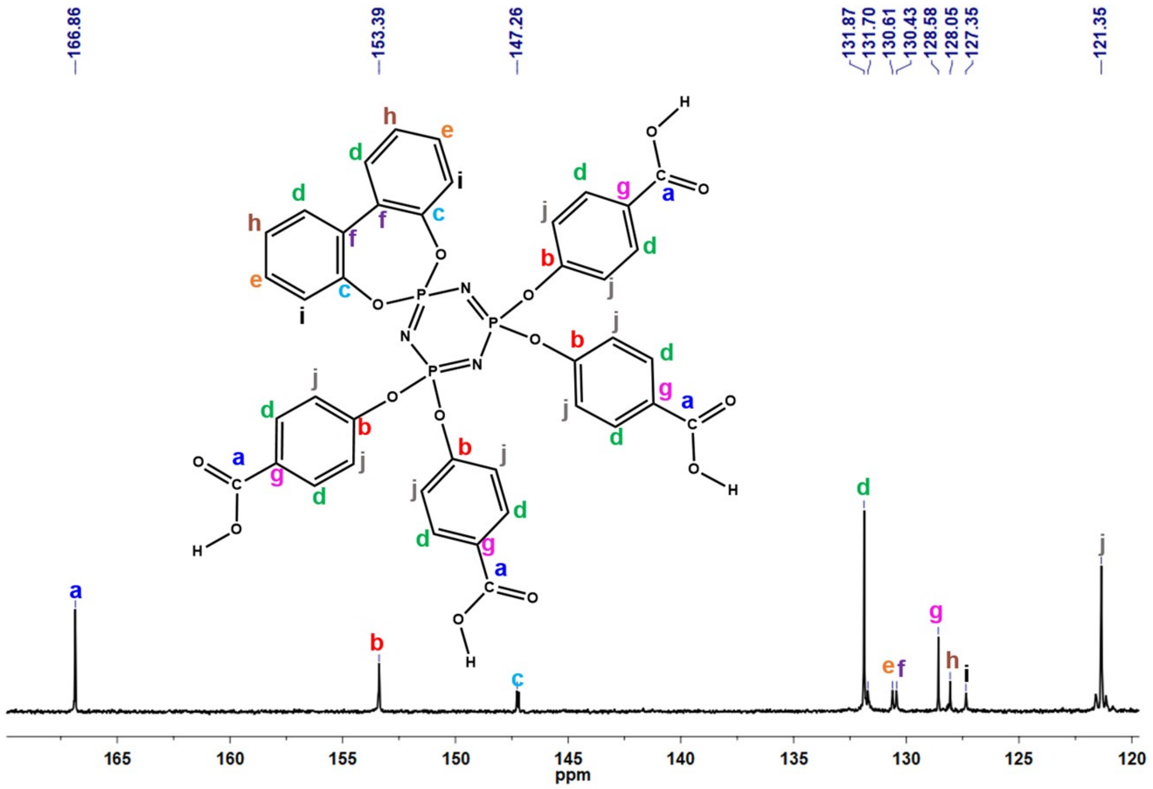

Fig. S14 ^{13}C-NMR spectrum of compound $\mathbf{L_2}$.

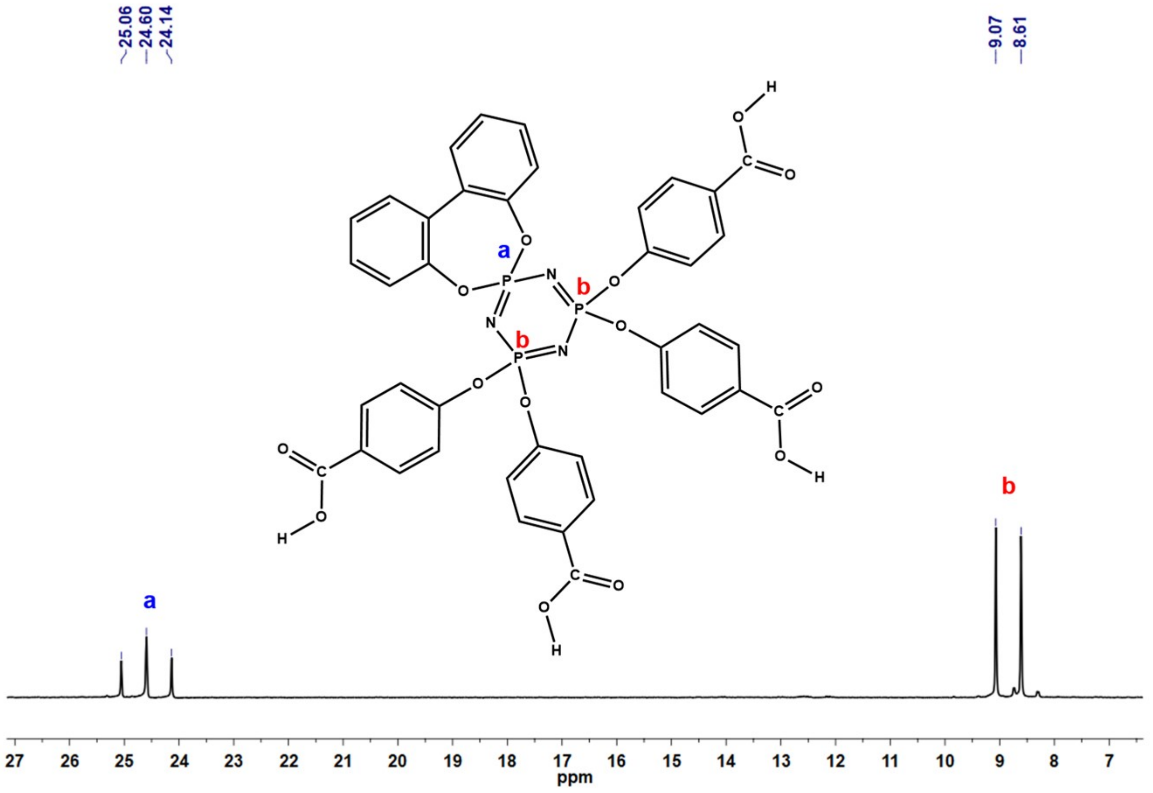

Fig. S15 ^{31}P-NMR spectrum of compound $\mathbf{L_2}$.

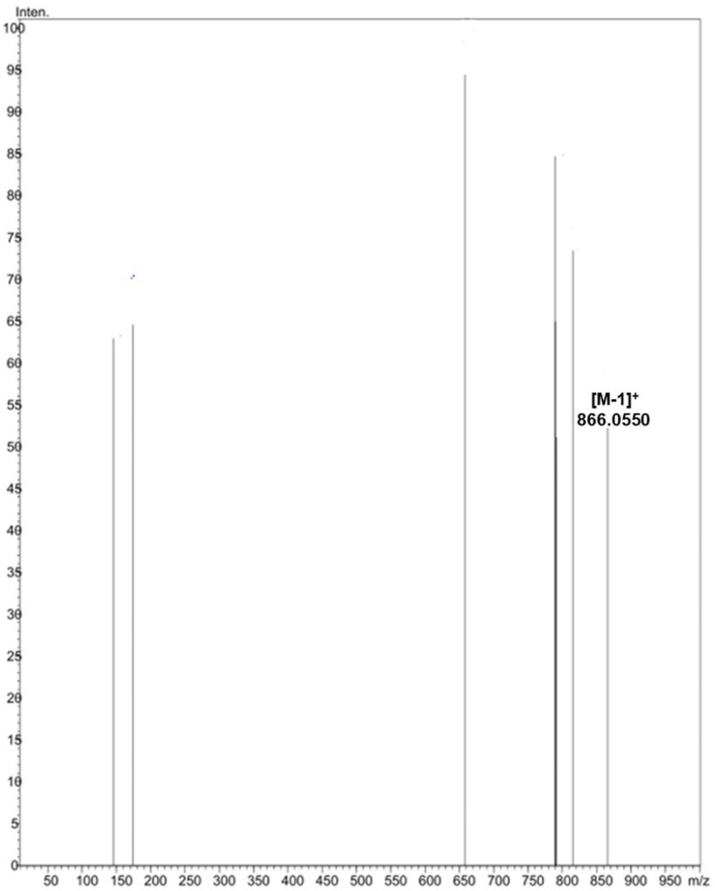

Fig. S16 ESI-MS of compound $\mathbf{L_2}$.

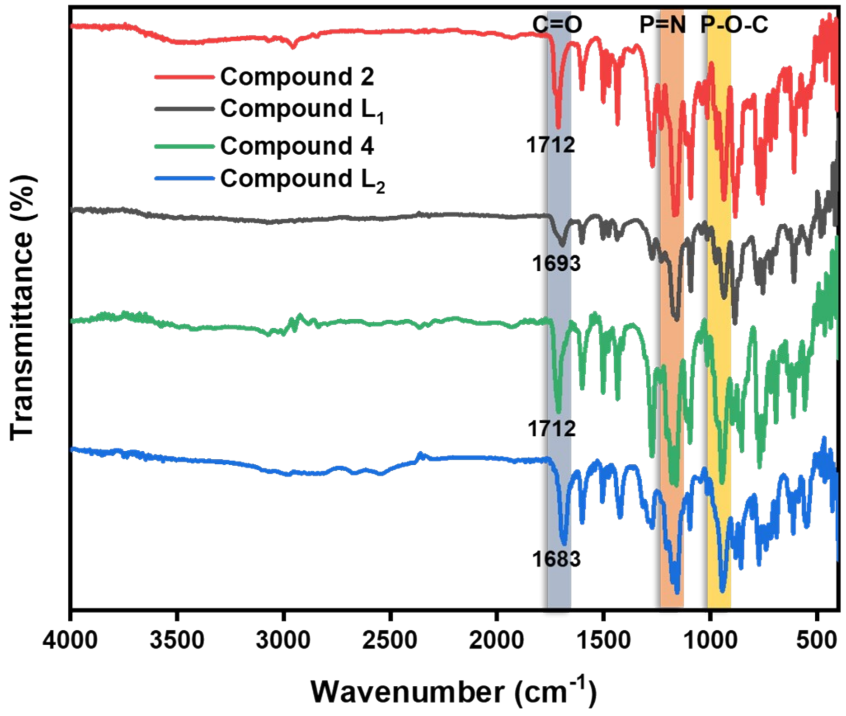

Fig. S17 FT-IR spectra of compounds **2**, $\mathbf{L_1}$, **4** and $\mathbf{L_2}$

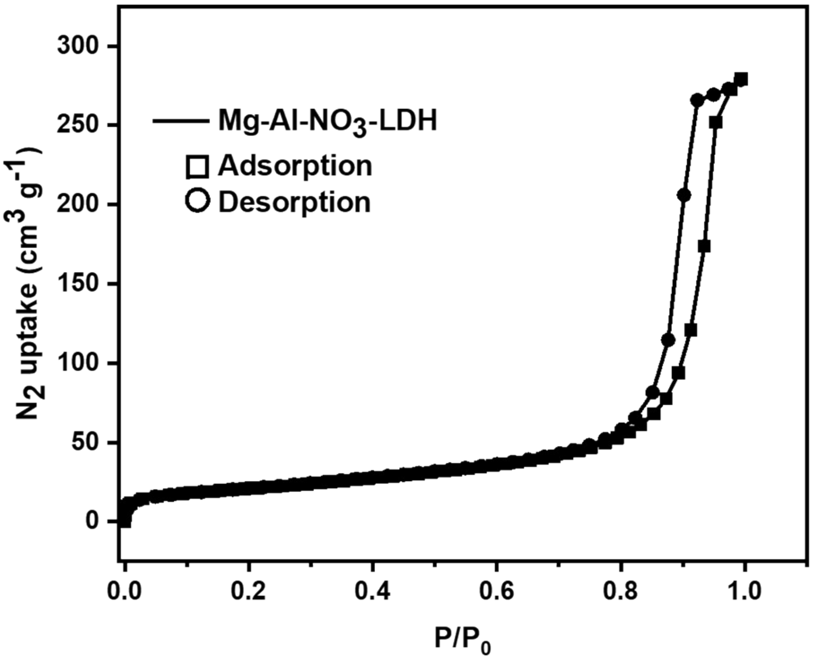

Fig. S18 N_2 adsorption and desorption isotherm for Mg-Al-NO_3-LDH.

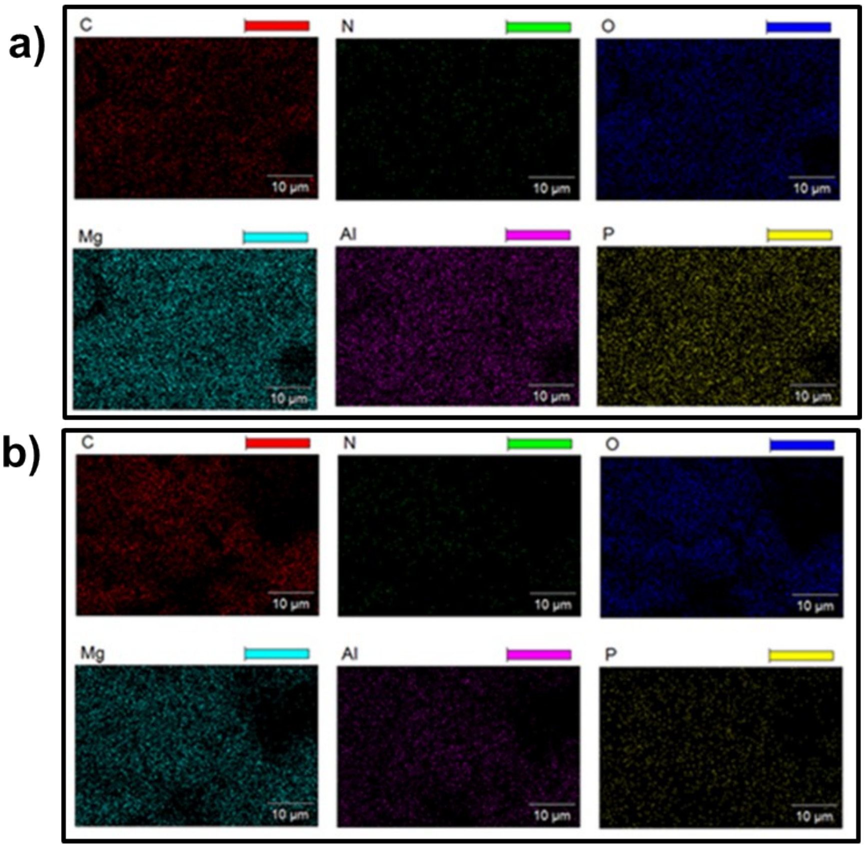

Fig. S19 Elemental mapping analysis of C, N, O, Mg, Al, P of a) LDH-PN-TC, b) LDH-PN-HC

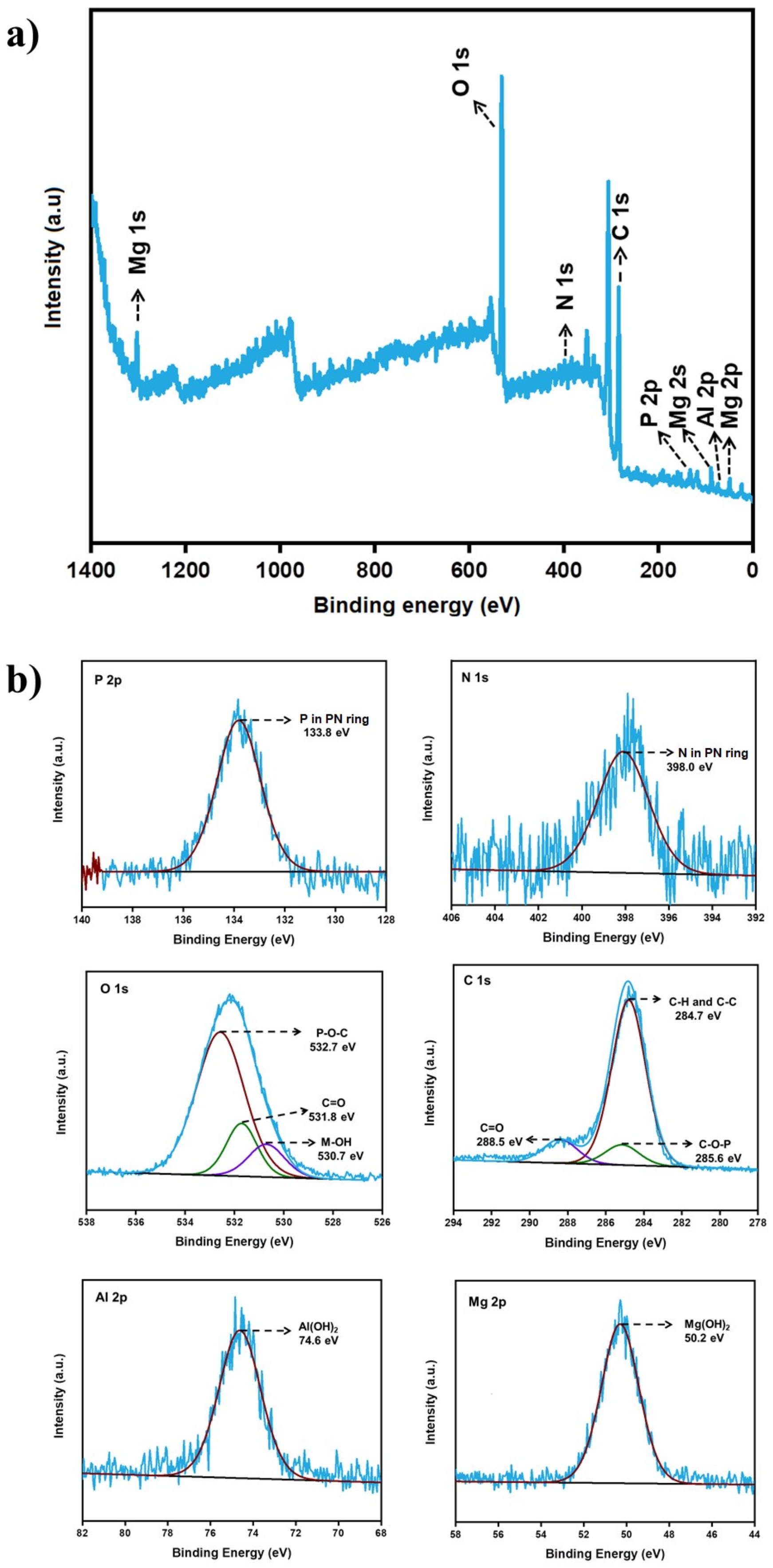

Fig. S20 (a) XPS survey spectrum of LDH-PN-TC; (b)) P 2p, N 1s, O 1s, C 1s, Al 2p and Mg 2p High-resolution spectra of LDH-PN-TC.

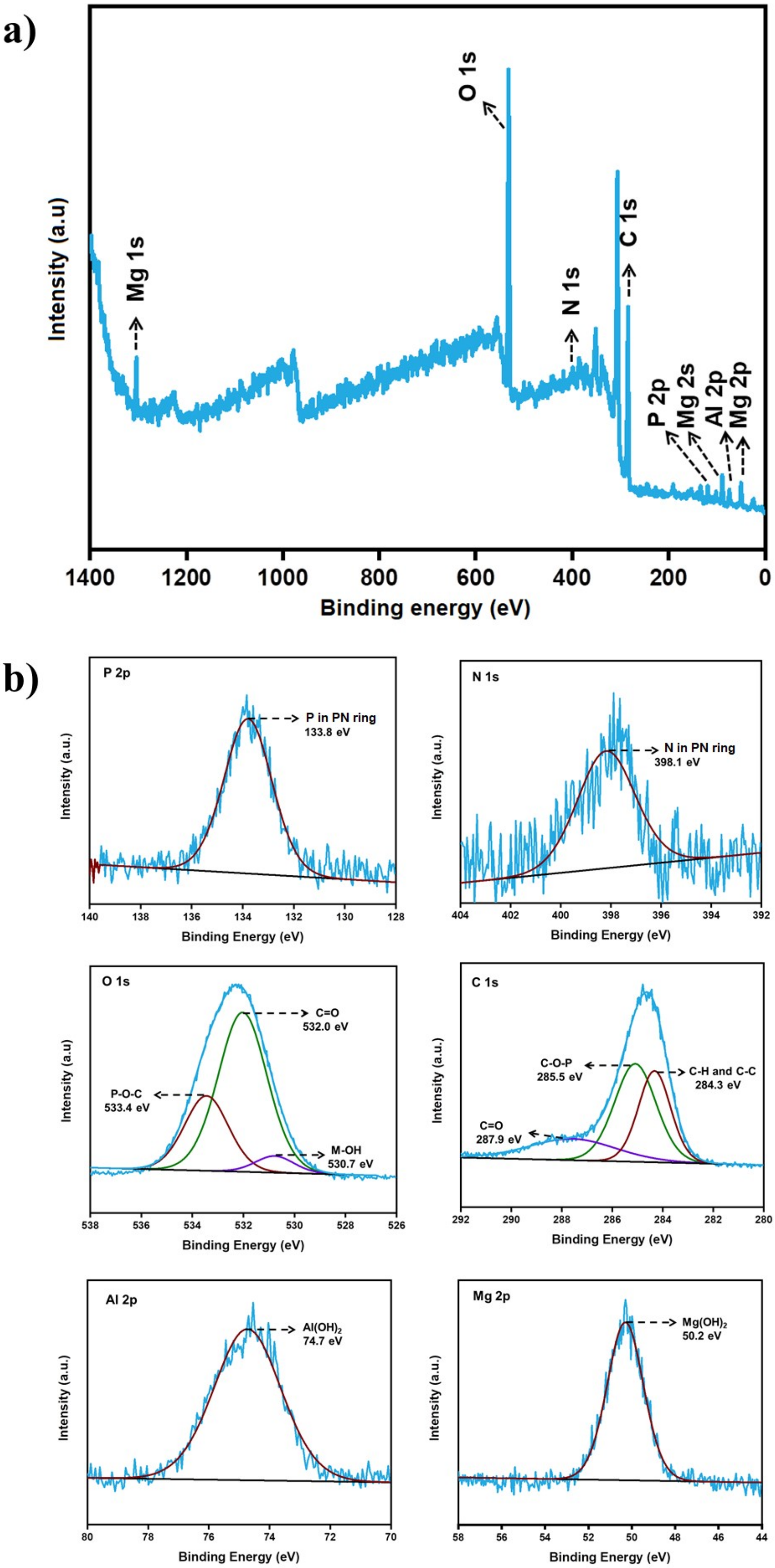

Fig. S21 (a) XPS survey spectrum of LDH-PN-HC; (b)) P 2p, N 1s, O 1s, C 1s, Al 2p and Mg 2p High-resolution spectra of LDH-PN-HC.

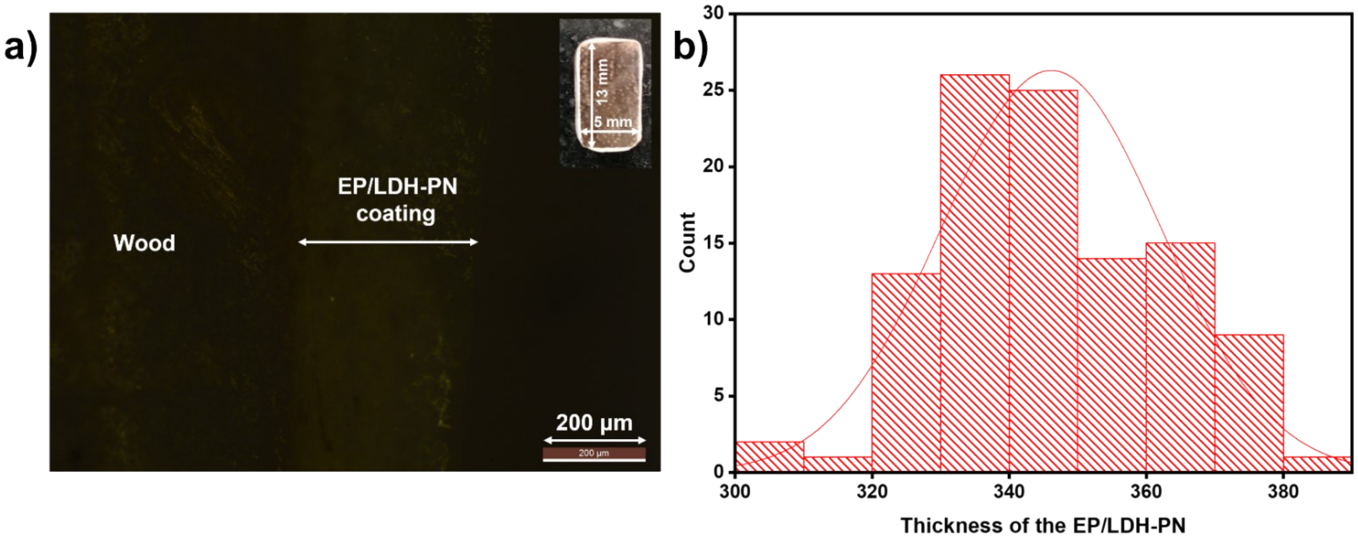

Fig. S22 (a) A microscopic image of the wood and EP/LDH-PN composite coated wood substrate and the inset shows a cross-sectional image of EP/LDH-PN composite coated wood substrate; (b) Histogram diagram for the calculation of average thickness of the EP/LDH-PN composite.

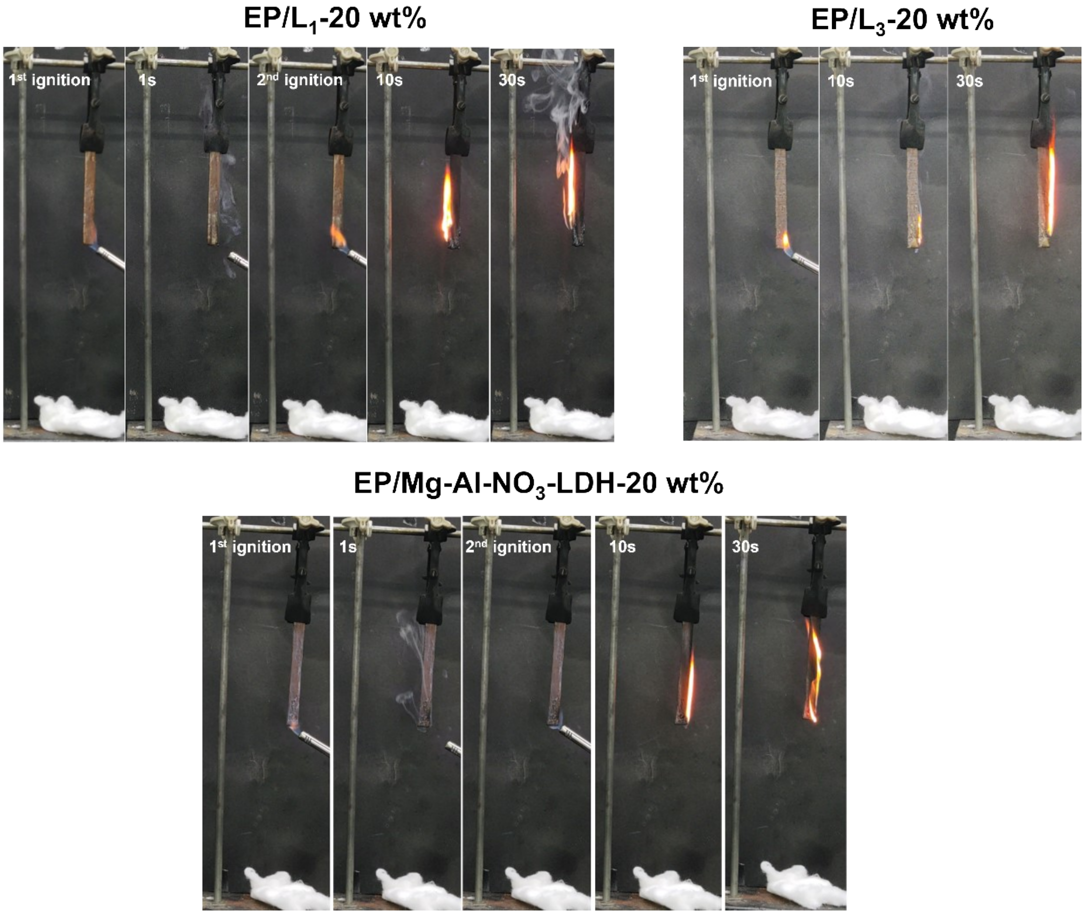

Fig. S23. Digital photos of EP/L_1-20 wt%, EP/L_3-20 wt%, EP/Mg-Al-NO_3-LDH-20 wt% composite coated wood substrates during the UL94 vertical burning process.
